# Identification of a six microRNA signature as a novel potential prognostic biomarker in patients with head and neck squamous cell carcinoma

**DOI:** 10.18632/oncotarget.7781

**Published:** 2016-02-27

**Authors:** Hao Shi, Jian Chen, Yuncheng Li, Guojun Li, Rong Zhong, Dandan Du, Ruiwei Meng, Weijia Kong, Meixia Lu

**Affiliations:** ^1^ Department of Epidemiology and Biostatistics, and The Ministry of Education Key Laboratory of Environment and Health, School of Public Health, Tongji Medical College, Huazhong University of Science and Technology, Wuhan, Hubei, China; ^2^ Department of Otorhinolaryngology, Union Hospital of Tongji Medical College, Huazhong University of Science and Technology, Wuhan, Hubei, China; ^3^ Department of Head and Neck Surgery, Hubei Cancer Hospital, and Hubei Key Laboratory of Medical Information Analysis & Tumor Diagnosis and Treatment, Wuhan, Hubei, China; ^4^ Department of Head and Neck Surgery, University of Texas MD Anderson Cancer Center, Houston, Texas, USA

**Keywords:** HNSCC, miRNA, prognosis, overall survival, TCGA

## Abstract

The 5-year survival rate of patients with head and neck squamous cell carcinoma (HNSCC) was only 40%-50%. To investigate the prognostic and predictive value of specific mircoRNAs (miRNAs) in HNSCC. We identified 19 miRNAs associated with over survival (OS) of patients with HNSCC in different clinical classes between 492 HNSCC tissues and 44 normal tissues from The Cancer Genome Atlas (TCGA) dataset. A signature of six miRNAs was identified by the supervised principal component method in the training set. The AUC of the ROC curve for the six microRNA signature predicting 5-year survival was 0.737 (95%CI, 0.627-0.825) in the testing set and 0.708 (95%CI, 0.616-0.785) in the total dataset. In the multivariate Cox regression analysis, patients with high-risk scores had shorter OS (HR, 2.380, 95%CI, 1.361-4.303) than patients with low-risk scores in the total dataset. Therefore, these results provided a new prospect for prognostic biomarker of HNSCC.

## INTRODUCTION

Head and neck squamous cell carcinoma (HNSCC) originates from the upper aerodigestive tract including oral cavity, oropharynx, hypopharynx and larynx, which is the sixth most common cancer. Approximately 600,000 new patients are diagnosed, and 350,000 patients died of this disease worldwide annually [1]. Furthermore, 5-year survival rate of patients with this disease was only 40 - 50% [2]. However, the prognosis of HNSCC patients has not any significantly improvement in the past decade [3]. Recent studies have found that tobacco use and HPV status in patients with HNSCC had significant prognostic importance [4].

MicroRNAs (miRNAs) are small non-coding RNAs of 18-25 nucleotides in length that regulate gene expression by binding to the 3′-untranslated region (3′-UTR) of their target mRNAs, resulting in mRNA degradation and/or inhibition of mRNA translation [5]. MiRNAs play a critical role in many processes such as cellular proliferation, apoptosis, migration, and differentiation [6-8]. Studies had previously found that some circulating miRNAs strongly associates with the invasion, and metastasis of cancer [9]. Therefore, circulating miRNAs may be a potential novel biomarkers for cancer detection and prognosis. For instance, a 4-miRNAs panel was associated with overall survival of patients with non-small cell lung cancer [10]. In breast cancer, higher levels of circulating hsa-miR-122 can predictively indicate metastatic recurrence in patients with stage II and stage III [11]. Hsa-miR-25 is associated with poor survival of gastric cancer patients by inhibiting transducer of ERBB2 [12]. Hsa-miR-7 expression is associated with poor prognosis via EGFR regulation in colorectal cancer [13]. Other studies showed that high expression of hsa-miR-19a and hsa-miR-21 and low expression of hsa-miR-375 were associated with shorter overall survival in laryngeal squamous cell carcinoma patients [14-16].

However, most of studies were based on a small number of patients, or inconsistent in these sets of miRNA markers due to the heterogeneous of disease and variations in the approaches for selection of miRNAs. The Cancer Genome Atlas (TCGA) released a large number of miRNA sequencing data for HNSCC patients. Therefore, the aim of this study was thus to assess systematically the predictive value of specific miRNA signature for 5-year survival of patients with HNSCC from a large dataset of TCGA.

## RESULTS

### Characters of the datasets

A total of 525 miRNA expression profiles (level 3 data) were obtained from TCGA. In miRNA tumor profiles, the expression of 1046 human miRNAs in HNSCC samples was assessed using the Illumina HiSeq Systems (n = 488) and Genome Analyzer (n = 37). MiRNA expression profiles for normal tissues (n = 44) were also analyzed using the Illumina HiSeq System. The clinical data for those patients obtained from TCGA were available. According to included criteria, a total of 492 HNSCC patients were finally enrolled in the study. For subsequent analysis, we randomly divided the total patients into the training set (n=246) and testing set (n=246) respectively. There was no significant difference on the clinical covariates between the two sets (*P* > 0.05). (Table 1)

**Table 1:** Clinical covariates for the TCGA HNSCC cohort

Covariates		Total n=492	Training set n=246	Testing set n=246	*P*-value
Age, years, no (%)	<=65	314(63.82)	152(61.79)	162(65.85)	0.398^a^
	>65	178(36.18)	94(38.21)	84(34.15)	
Gender, no (%)	Male	357(72.56)	176(71.55)	181(73.58)	0.686^a^
	Female	135(24.44)	70(28.45)	65(26.42)	
Clinical N, no (%)	N0	236(49.06)	120(49.59)	116(48.54)	0.750^b^
	N1	78(16.22)	41(16.94)	37(15.48)	
	N2	158(32.85)	78(32.23)	80(33.47)	
	N3	9(1.87)	3(1.24)	6(2.51)	
Clinical M, no (%)	M0	470(98.95)	235(98.74)	235(99.16)	1^b^
	M1	5(1.05)	3(1.26)	2(0.84)	
Clinical T, no (%)	T1	30(6.20)	11(4.55)	19(7.85)	0.073^a^
	T2	144(29.75)	83(34.30)	61(25.21)	
	T3	134(27.69)	60(24.79)	74(30.58)	
	T4	176(36.36)	88(36.36)	88(36.36)	
Clinical Stage, no (%)	I	18(3.70)	6(2.47)	12(4.94)	0.171^a^
	II	94(19.34)	55(22.63)	39(16.05)	
	III	100(20.58)	48(19.75)	52(21.40)	
	IV	274(56.38)	134(55.15)	140(57.61)	
Smoking status, no (%)	Non-smoker	114(23.75)	58(24.27)	56(23.24)	0.893^a^
	Reformed smoker	206(42.92)	100(41.84)	106(43.98)	
	Current smoker	160(33.33)	81(33.89)	79(32.78)	
Alcohol, no (%)	Yes	332(68.74)	169(69.83)	163(67.63)	0.672^a^
	No	151(31.26)	73(30.17)	78(32.37)	
HPV status^#^, no (%)	Positive	39(36.11)	22(44)	17(29.31)	0.166^a^
	Negative	69(63.89)	28(66)	41(70.69)	
Lymphnodes positive^*^, no (%)	Yes	225(58.29)	111(58.42)	114(58.16)	1^a^
	No	161(41.71)	79(41.58)	82(41.84)	
Perineural invasion present, no (%)	Yes	168(48.14)	88(48.89)	80(47.34)	0.855^a^
	No	181(51.86)	92(51.11)	89(52.66)	
Pathologic T, no (%)	T1	41(9.56)	19(8.84)	22(10.28)	0.545^a^
	T2	128(29.84)	64(29.77)	64(29.91)	
	T3	94(21.91)	53(24.65)	41(19.16)	
	T4	166(38.69)	79(36.74)	87(40.65)	
Pathologic N, no (%)	N0	163(41.37)	80(40.82)	83(41.92)	0.512^a^
	N1	64(16.24)	36(18.37)	28(14.14)	
	N2-3	167(42.39)	80(40.82)	87(43.94)	
Pathologic Stage, no (%)	I	23(5.45)	14(6.54)	9(4.33)	0.313^a^
	II	72(17.06)	32(14.95)	40(19.23)	
	III	73(17.30)	42(19.63)	31(14.90)	
	IV	254(60.19)	126(58.88)	128(61.54)	
Tumor grade, no (%)	G1	51(10.81)	27(11.59)	24(10.04)	0.419^a^
	G2	295(62.5)	150(64.38)	145(60.67)	
	G3-4	126(26.69)	56(24.03)	70(29.29)	
Survival time, month (mean±sd)		23.38±28.04	23.42±26.55	23.33±29.50	0.973^c^
Vital status, no (%)	Alive	328(66.67)	173(70.33)	155(63.01)	0.104^a^
	Dead	164(33.33)	73(29.67)	91(36.99)	

# HPV status positive by p16 testing;

* Lymphnodes positive by HE;

a *x^2^* test;

b Fisher's exact test;

c Student's t-test

### Identification of differentially expressed miRNAs in HNSCC patients

Analysis of miRNA expression profiles in HNSCC patient tissues (n = 492) compared with normal tissues (n = 44) identified a total of 98 differentially expressed miRNAs (logFC > 1 or logFC < −1, *P* < 0.05 after FDR adjustment). Of these, 55 miRNAs were overexpressed, and 43 miRNAs were downexpressed. Among the overexpressed miRNAs, three miRNAs (hsa-miR-105-1, hsa-miR-105-2, and hsa-miR-767) exhibited over 5-fold increased expression, while hsa-miR-381, hsa-miR-1-2, hsa-miR-449a, and hsa-miR-375 exhibited over -3-fold decreased expression among 43 downexpressed miRNAs (Supplementary Table S1).

### Association of miRNAs expression and clinical features with OS of HNSCC patients

We conducted univariate Cox regression between clinical covariates and HNSCC to confirm the prognostic significance of the clinical covariates. After the analysis, clinical variables of age, smoking status, lymphnodes positive, perineural invasion present, pathologic N stage, pathologic T stage, pathologic disease stage, and tumor grade were significantly associated with OS. However, we did not find other clinical variables of gender, clinic N stage, clinic T stage, clinic M stage, clinic disease stage, HPV status, and alcohol history significantly associated with OS. Kaplan-Meier survival curves and log rank test for these variables were shown in Supplementary Figure S1–Figure S15.

**Figure 1:**
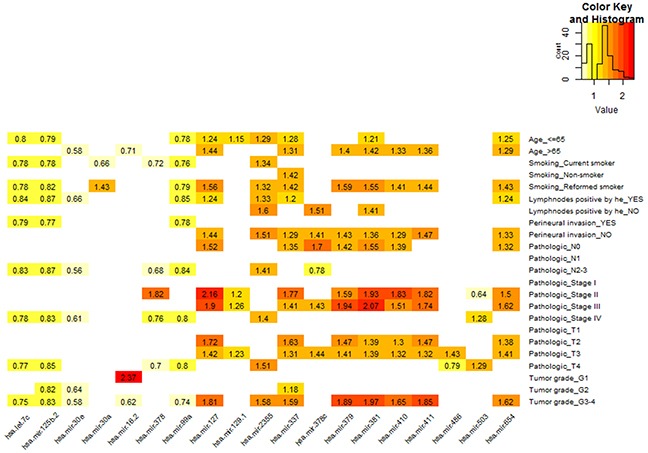
MiRNAs associated with prognosis in different clinical subclasses of TCGA HNSCC cohort The matrix visualizes the significant HRs for the 19 miRNAs in TCGA HNSCC cohort. The HRs were for expression which on a log2 scale with significant univariate Cox regression (*P* < 0.05).

Then, we conducted univariate Cox regression to identify common miRNAs associated with OS within each of the following independent classes: age, smoking status, lymphnodes positive, perineural invasion present, pathologic N stage, pathologic T stage, pathologic disease stage, and tumor grade. Within each subset of clinical characteristics, the patient subclasses represented non-overlapping sets, respectively. MiRNAs were selected as candidate markers if they were associatedsignificance with OS in at least two independent categories for each covariate. The respective HRs for the association of miRNA with OS in each subclass were shown in Figure 1. A total of 19 miRNAs were identified in this analysis.

### Definition of miRNA prognostic model

We selected six miRNAs by the supervised principal component method in the training set. And then, we developed a miRNA prognostic model. The miRNAs expression level was as the log2 reads per million of total aligned miRNA reads. The prognostic score was calculated as follows: Prognostic-score = (−1.155×expression level of hsa-let-7c) + (−1.063×expression level of hsa-miR-125b-2) + (1.217×expression level of hsa-miR-129-1) + (1.137×expression level of hsa-miR-337) + (1.013×expression level of hsa-miR-654) + (−1.157×expression level of hsa-miR-99a). We classified the samples into high risk or low risk group using the best cutoff point of miRNA scores with optimum sensitivity and specificity according to ROC curve for predicting 5-year survival in the training set. The cutoff point was −16.070 with 71.48% sensitivity and 70.20% specificity.

### Prognostic value of the six microRNA signature in HNSCC

**Figure 2:**
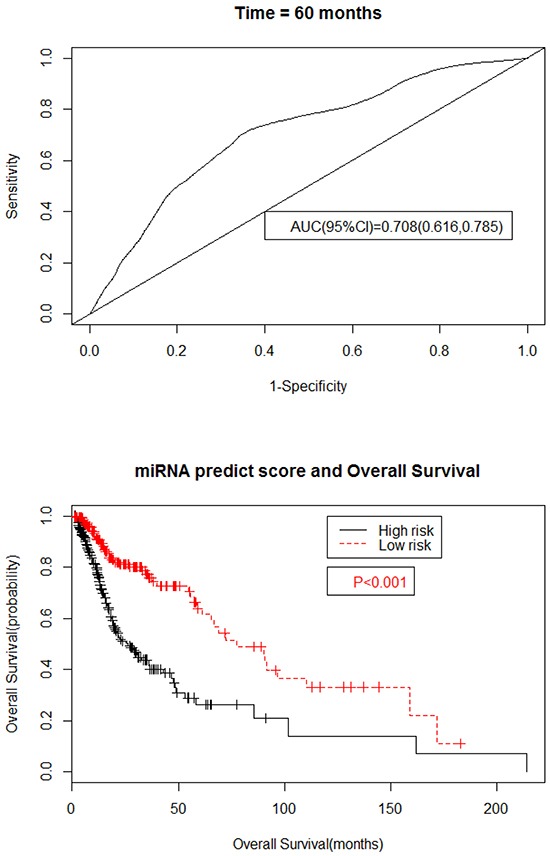
Kaplan–Meier and ROC curves for the six microRNA signature in TCGA HNSCC cohort **A.** The ROC curve for predicting 5-year survival had an AUC of 0.708 (95%CI, 0.616-0.785). The 95%CI of AUC were calculated from 1000 bootstrap of the survival data. **B.** The Kaplan–Meier curves for HNSCC risk groups obtained from the TCGA cohort divided by the cutoff point. The P value of the log-rank test was <0.01.

The six microRNA signature showed greater predicting prognosis capacity for predicting 5-year survival in HNSCC with an AUC of 0.737 (95%CI, 0.627-0.825) in the testing set (Supplementary Figure S16A) and an AUC of 0.708 (95%CI, 0.616-0.785) in the total HNSCC patients (Figure 2A), respectively.

**Figure 3:**
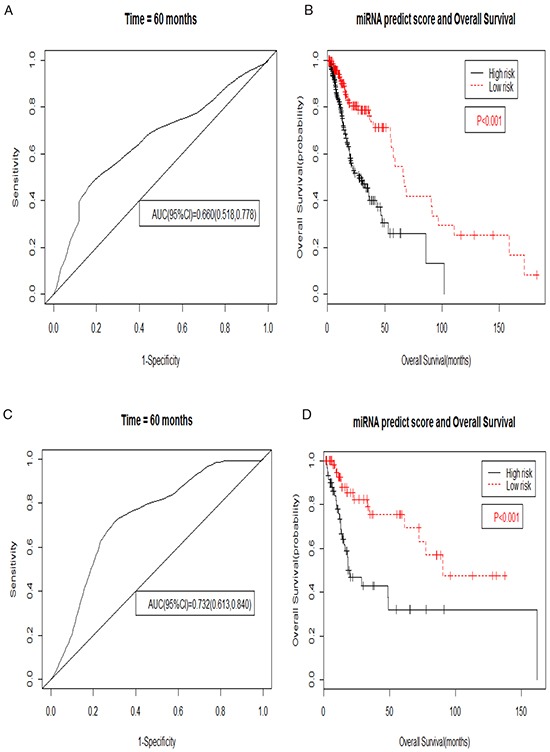
Kaplan–Meier and ROC curves for the six microRNA signature in TCGA HNSCC tumor grade group **A.** The ROC curve for predicting 5-year survival had an AUC of 0.660 (95%CI, 0.518-0.778) in the G1-2 group. The 95%CI of AUC were calculated from 1000 bootstrap of the survival data. **B.** The Kaplan–Meier curves for HNSCC risk groups obtained from the TCGA cohort G1-2 group divided by the cutoff point. The P value of the log-rank test was <0.01. **C.** The ROC curve for predicting 5-year survival had an AUC of 0.732 (95%CI, 0.613-0.840) in the G3-4 group. The 95%CI of AUC were calculated from 1000 bootstrap of the survival data. **D.** The Kaplan–Meier curves for HNSCC risk groups obtained from the TCGA cohort G3-4 group divided by the cutoff point. The P value of the log-rank test was <0.01.

We then evaluated the six microRNA signature on the each subgroup of clinical characteristics The six microRNA signature was not significantly predictive for predicting 5-year survival only in the pathologic T4 group (Supplementary Figure S19C) and were significantly in the remaining group (Figure 3A/3C and Supplementary Figure S17A/C, Figure S18A/C and Figure S19A). The OS rate of patients with low risk group was significantly higher than that of patients with high risk group in the all subgroup (Figure 3B/3D and Supplementary Figure S17B/D - Figure S19B/D).

Compared with patients with low-risk scores, patients with high-risk scores in the TCGA HNSCC cohort had significantly shorter OS (HR, 2.380, 95%CI, 1.361-4.303) after adjusted age, perineural invasion, pathologic T stage, pathologic N stage by multivariate Cox proportional hazards regression analysis according to the backward stepwise method of screening variables in Table 2.

**Table 2:** Multivariate cox proportional hazards analysis

Items	Coefficient	P-value	HR^#^	HR(95%CI^*^)	
				Lower	Upper
Age (>65 vs. <=65)	0.505	0.051	1.656	0.998	2.748
Perineural invasion present (Yes vs. No)	0.559	0.050	1.750	1.000	3.060
Pathologic T (T2 vs. T1)	−0.785	0.343	0.456	0.090	2.306
Pathologic T (T3 vs. T1)	0.145	0.851	1.156	0.255	5.249
Pathologic T (T4 vs. T1)	0.275	0.716	1.316	0.301	5.762
Pathologic N (N1 vs. N0)	0.038	0.926	1.039	0.467	2.309
Pathologic N (N2-3 vs. N0)	0.660	0.029	1.936	1.072	3.497
MiRNA model scores (High vs. Low)	0.867	0.004	2.380	1.316	4.303

# Hazard ratio;

* Confidence interval

### Target prediction and functional enrichment of the six microRNA signature in HNSCC

**Figure 4:**
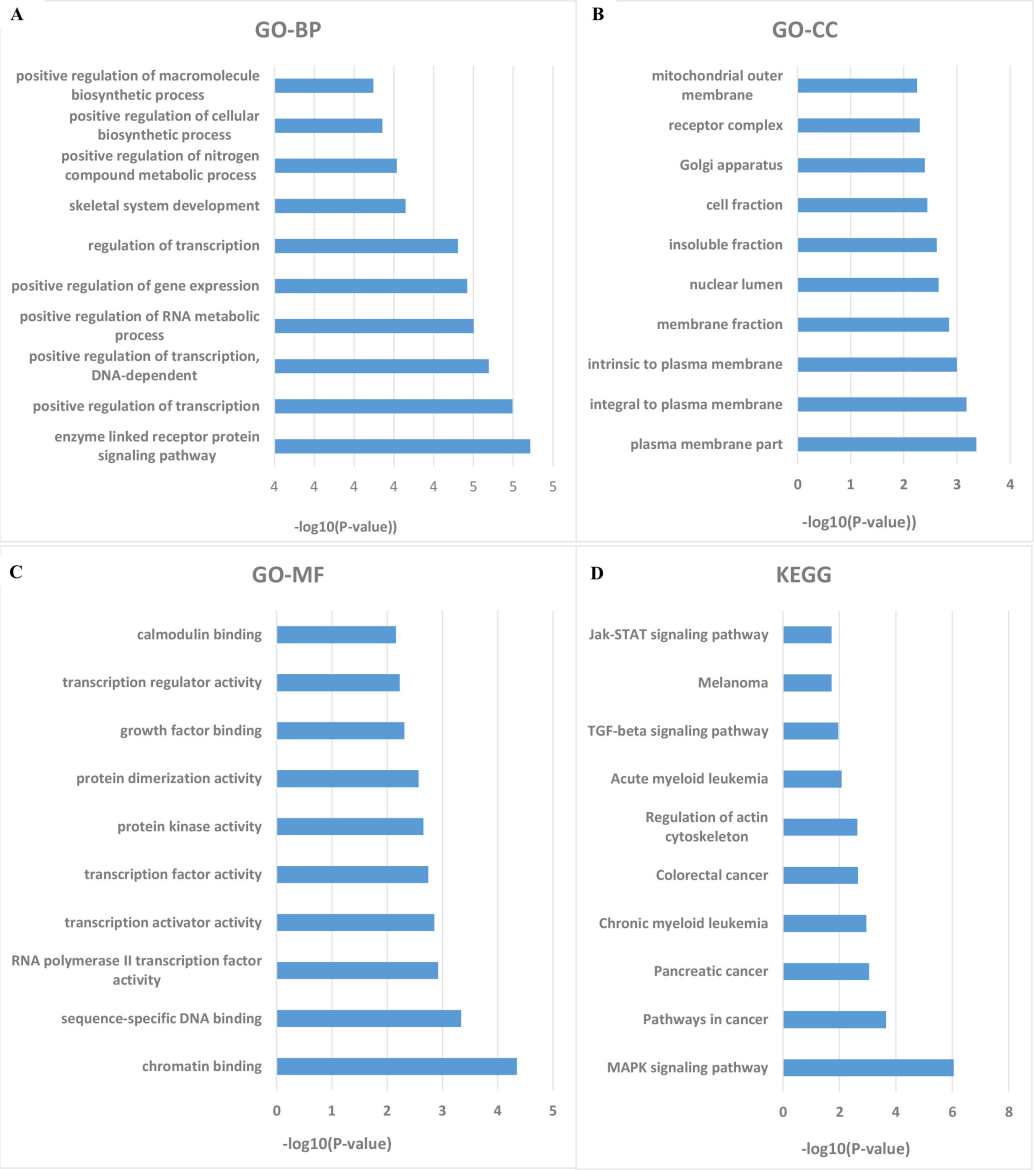
The top ten of GO term and pathway by target genes of six microRNA signature in TCGA HNSCC cohort Significant analysis was determined when P values were corrected for false discovery rate (FDR). **A.** Parts of GO BP categories of six microRNA signature. **B.** Parts of GO CC categories of six microRNA signature. **C.** Parts of GO MF categories of six microRNA signature. **D.** Parts of KEGG pathway of six microRNA signature.

The numbers of the target genes of the six miRNAs were 8437, 7253, and 1119, which were predicted by three data sets using miRanda, Targetscan, and PicTar programs, respectively. A total of 314 target genes were included in the three data sets (Supplementary Figure S20). We performed enrichment analyses to elucidate the biological function of target genes of the six microRNA signature. Finally, Gene ontology (GO) analysis revealed that there were 144 of the proteins were associated with biological process (BP), 37 of the proteins with cellular component (CC), and 31 of the proteins with molecular function (MF), respectively. The top ten enriched functional analysis was shown in Figure 4. The top enriched biological process was enzyme linked receptor protein signaling pathway. The top enriched cellular component and molecular function was plasma membrane part and chromatin binding, respectively. Therefore, a total of 23 KEGG pathways were enriched by the six microRNA signature. The top enriched KEGG pathway was the MAPK signaling pathway. The top 15 functional enrichment of target genes for six microRNA signature were summarized in the Supplementary Table S2.

## DISCUSSION

MiRNAs have been reported in all stages of neoplastic progression including apoptosis, proliferation, progression, metastasis, invasion, and relapse [17]. Recent studies have found that miRNAs were associated with lymph node metastasis, T classification, clinical stage, and prognosis in patients with HNSCC [18-20]. Shen et al. found that the expression of hsa-miR-34a was positively correlated with survival rate and could independently predict survival in patients with laryngeal squamous cell carcinoma [21]. Hsa-miR-203 may be used as a predictive marker of survival in laryngeal squamous cell carcinoma patients because the expression of hsa-miR-203 was associated with lymph node metastasis, advanced clinical stages, and decreased 5-year survival rate [22, 23]. Hou et al. found that circulating hsa-miR-99a may be used as biomarkers to assess the efficacy of therapy and the prognosis of HNSCC [24]. Therefore, we applied a miRNAs signature as a potential biomarker for predicting survival in HNSCC using TCGA datasets.

In the present study, we have identified a six microRNA signature consisting of hsa-let-7c, hsa-miR-125b-2, hsa-miR-129-1, hsa-miR-337, hsa-miR-654, and hsa-miR-99a, which was validated as an independent predictor for HNSCC patient survival. The AUC of the ROC curve for the six microRNA signature predicting 5-year survival was 0.737 (95%CI, 0.627-0.825) in the testing set and 0.708 (95%CI, 0.616-0.785) in the total dataset. The six microRNA signature have a good performance for predicting 5-year survival in HNSCC.

Among the six miRNAs, low expression of hsa-miR-99a was associated with poor prognosis in patients with HNSCC. On the contrary, overexpression of hsa-miR-99a distinctly inhibited cell proliferation and induced apoptosis by down-regulating the expression level of IGF1R and mTOR genes in oral cancer cells [25-27]. Accordingly, overexpression of IGF1R and activation of mTOR signaling path have been observed in HNSCC, and were often associated with poor prognosis [28]. Hsa-miR-125b controls the apoptotic pathway by targeting the BAK1 gene in HNSCC [29]. Down-regulated hsa-let-7c was significantly associated with metastasis and poor survival of patients, and hsa-let-7c inhibits cancer metastasis by degrading ITGB3 and MAP4K3 in non-small cell lung cancer [30]. Hsa-let-7c was down-regulated in HNSCC [31, 32]. To date, to our knowledge, this is the first to report the association of hsa-miR-129-1, hsa-miR-337, and hsa-miR-654 with OS in HNSCC.

The carcinogenic process of HNSCC is a multi-step process driven by a series of genetic and epigenetic alterations in tumor suppressor genes and oncogenes resulting in the progression from a normal cell to a cancer cell [33]. Through the enrichment and function analysis of DAVID database, we found that the target genes of these miRNAs may participate in many important biological processes, including regulation of transcription, positive regulation of transcription, and positive regulation of gene expression. In the KEGG pathway analysis, these target genes also take part in many important signaling pathways such as MAPK signaling pathway, cancer pathways, and TGF-beta signaling pathway. Aberrant activation of the p38 MAPK pathway may halt HNSCC cells growth and metastasis by reducing tumor-induced inflammation and angiogenesis [34]. TGF-β signaling causes the pro-proliferative and tumor-suppressive responses according to the biological context [35]. SMAD4 alterations may explain the decreased tumor suppressive effect of TGF-β signaling in HNSCC [36, 37].

There were a number of limitations in this study. Firstly, the data in this study was from a single source (TCGA) and randomly assigning samples to training set and testing set for the development and assessment of the prognostic model. The performance of these miRNAs signature would be more reliable if validation is performed in an independent external data sets with long-term follow up. Secondly, the TCGA HNSCC cohort had a relatively high censored rate, which may affect the reliability of the Kaplan-Meier estimates.

In conclusion, our results showed that the six microRNA signature significantly predicts the 5-year survival of HNSCC patients in the TCGA cohort, indicating that it may be a novel potential biomarker for prognosis of HNSCC. This finding requires further confirmation in independent larger cohorts in future studies.

## MATERIALS AND METHODS

### Patient characteristics and miRNA dataset in TCGA

The miRNA expression profiles (level 3 data) and corresponding clinical data for HNSCC patients were obtained from TCGA data portal (January 2013, https://tcga-data.nci.nih.gov/tcga/tcgaHome2.jsp). Both the miRNA profiles data and clinical data of HNSCC are publically available and open-access. These patients' extended demographics were characterized by the TCGA. The patients were included in the study to meet the following criteria: (1) patients with fully characterized (clinical data and miRNA profiles) tumors; (2) patients with at least 1 month of over survival (OS).

### Identification of differentially expressed miRNAs between HNSCC and normal tissue

To identify miRNAs differentially expressed between HNSCC and normal tissues, the raw counts of miRNA expression obtained from the TCGA dataset (492 HNSCC samples and 44 normal tissue) were normalized by a weighted trimmed mean of the log expression ratios (TMM, Trimmed mean of M values method [38]). One miRNA expression filter was miRNAs expressed in at least two normal or tumor samples and with at least 100 counts per million in the profile. The batch effect was removed using a generalized linear model (GLM) [39]. The expression differences were characterized by logFC (log 2 fold change) and associated P- values. The logFC >1 and logFC < −1 with FDR-adjusted (FDR adjust: Benjamini & Hochberg) *P* < 0.05 respectively represented up-regulated and down-regulated miRNAs. The analysis was performed using the R/Bioconductor package of edgeR [40].

### Survival analysis

Clinical covariates for HNSCC patients are summarized in Table 1. The univariate Cox regression was performed to test the association between clinical covariates and OS. The equality for survival distributions in different groups were test by the Kaplan-Meier and log rank method. The miRNA expression level was as the log2 reads per million of total aligned miRNA reads. Univariate Cox regression was used to identify common miRNAs related to OS within each of clinical characteristics that were significantly associated with OS. Within each group of clinical characteristics, the patient subclasses represented non-overlapping sets. Common miRNAs associated with OS in at least two independent categories for each covariate were selected as candidate markers, using a P-value of 0.05 as the cutoff for miRNA selection. The hazard ratio was the ratio of hazards for a twofold change in the gene expression level. All reported P values were two-sided. The analysis was performed using R Packages of survival.

### Definition of prognostic model and ROC curve

The miRNAs selected as candidate markers under survival analysis were conducted to the supervised principal component analysis [41]. 10-fold cross-validation was used to estimate the optimal feature threshold in supervised principal components. The threshold of 10-fold cross-validation is equal to 1 serving as the optimal feature threshold in supervised principal components. The gene weights for the linear miRNA risk predictor were computed using the supervised principal component method. We used the linear miRNA prognostic model obtained from the training set to calculate a miRNAs signature prognostic score for each of 492 patients. We chose the best cutoff values with optimum sensitivity and specificity of the miRNAs signature prognostic scores to divide the patients into the high risk or low risk group in the ROC curve for predicting 5-year survival of the training set. Kaplan-Meier curves were used to estimate the survival for patients with high risk scores or low risk scores. In addition, the prognostic value of the miRNAs signature for 5-year survival of patients was also assessed in different clinical characteristics including smoking status, pathologic T, pathologic disease stage, and tumor grade.

The prognostic performance was measured using time-dependent receiver operating characteristic (ROC) curves. Since the majority of events occurred before 60 months, the ability of models to predict outcome at and around 60 months was assessed. Bootstrap 95%CI of AUC were calculated from 1000 bootstrap of the survival data. We used a multivariable analysis to evaluate the contribution of miRNAs as independent prognostic factors of patient survival. The backward stepwise method was employed to select the best model. All analysis were performed using R (Packages: survival, survivalROC, boot, and superpc).

### Target prediction and enrichment analysis

The target genes of miRNAs were predicted by three programs including miRanda, Targetscan, and PicTar. The target genes were selected by miRanda if the mirSVR score ≤ −0.1 and by Targetscan if the total context score ≤-0.1. The final target genes were selected, which were included in all the three data sets. The enrichment analysis of these target genes was analyzed using DAVID online analysis [42] (https://david.ncifcrf.gov/). The gene sets containing less than 5 genes overlapping were removed from the DAVID analysis, and analysis for significance was determined when P values were corrected for FDR.

## SUPPLEMENTARY FIGURES AND TABLES







## References

[1] Siegel R, Ma J, Zou Z, Jemal A (2014). Cancer statistics, 2014. CA Cancer J Clin.

[2] Leemans CR, Braakhuis BJ, Brakenhoff RH (2011). The molecular biology of head and neck cancer. Nature reviews Cancer.

[3] Kamangar F, Dores GM, Anderson WF (2006). Patterns of cancer incidence, mortality, and prevalence across five continents: defining priorities to reduce cancer disparities in different geographic regions of the world. Journal of clinical oncology.

[4] Ang KK, Harris J, Wheeler R, Weber R, Rosenthal DI, Nguyen-Tan PF, Westra WH, Chung CH, Jordan RC, Lu C, Kim H, Axelrod R, Silverman CC, Redmond KP, Gillison ML (2010). Human papillomavirus and survival of patients with oropharyngeal cancer. The New England journal of medicine.

[5] Carthew RW, Sontheimer EJ (2009). Origins and Mechanisms of miRNAs and siRNAs. Cell.

[6] Carleton M, Cleary MA, Linsley PS (2007). MicroRNAs and Cell Cycle Regulation. Cell Cycle.

[7] Chen C-Z (2005). MicroRNAs as Oncogenes and Tumor Suppressors. The New England journal of medicine.

[8] Bartel DP (2007). MicroRNAs: Genomics, biogenesis, mechanism, and function (Reprinted from Cell, vol 116, pg 281-297, 2004). Cell.

[9] Shen J, Stass SA, Jiang F (2013). MicroRNAs as potential biomarkers in human solid tumors. Cancer letters.

[10] Hu Z, Chen X, Zhao Y, Tian T, Jin G, Shu Y, Chen Y, Xu L, Zen K, Zhang C, Shen H (2010). Serum microRNA signatures identified in a genome-wide serum microRNA expression profiling predict survival of non-small-cell lung cancer. Journal of clinical oncology.

[11] Wu X, Somlo G, Yu Y, Palomares MR, Li AX, Zhou W, Chow A, Yen Y, Rossi JJ, Gao H, Wang J, Yuan YC, Frankel P, Li S, Ashing-Giwa KT, Sun G (2012). De novo sequencing of circulating miRNAs identifies novel markers predicting clinical outcome of locally advanced breast cancer. Journal of translational medicine.

[12] Li BS, Zuo QF, Zhao YL, Xiao B, Zhuang Y, Mao XH, Wu C, Yang SM, Zeng H, Zou QM, Guo G (2015). MicroRNA-25 promotes gastric cancer migration, invasion and proliferation by directly targeting transducer of ERBB2, 1 and correlates with poor survival. Oncogene.

[13] Suto T, Yokobori T, Yajima R, Morita H, Fujii T, Yamaguchi S, Altan B, Tsutsumi S, Asao T, Kuwano H (2015). MicroRNA-7 expression in colorectal cancer is associated with poor prognosis and regulates cetuximab sensitivity via EGFR regulation. Carcinogenesis.

[14] Wu H, Liu T, Wang R, Tian S, Liu M, Li X, Tang H (2011). MicroRNA-16 targets zyxin and promotes cell motility in human laryngeal carcinoma cell line HEp-2. IUBMB life.

[15] Hu A, Huang JJ, Xu WH, Jin XJ, Li JP, Tang YJ, Huang XF, Cui HJ, Sun GB (2014). miR-21 and miR-375 microRNAs as candidate diagnostic biomarkers in squamous cell carcinoma of the larynx: association with patient survival. American journal of translational research.

[16] Yu X, Wu Y, Liu Y, Deng H, Shen Z, Xiao B, Guo J (2014). miR-21, miR-106b and miR-375 as novel potential biomarkers for laryngeal squamous cell carcinoma. Current pharmaceutical biotechnology.

[17] Dhahbi JM (2014). Circulating small noncoding RNAs as biomarkers of aging. Ageing Research Reviews.

[18] Li G, Ren SL, Su ZW, Liu C, Deng TB, Huang DH, Tian YQ, Qiu YZ, Liu Y (2015). Increased expression of miR-93 is associated with poor prognosis in head and neck squamous cell carcinoma. Tumor Biol.

[19] Avissar M, McClean MD, Kelsey KT, Marsit CJ (2009). MicroRNA expression in head and neck cancer associates with alcohol consumption and survival. Carcinogenesis.

[20] Fletcher AM, Heaford AC, Trask DK (2008). Detection of Metastatic Head and Neck Squamous Cell Carcinoma Using the Relative Expression of Tissue-Specific Mir-205. Transl Oncol.

[21] Shen Z, Zhan G, Ye D, Ren Y, Cheng L, Wu Z, Guo J (2012). MicroRNA-34a affects the occurrence of laryngeal squamous cell carcinoma by targeting the antiapoptotic gene survivin. Medical oncology.

[22] Tian L, Li M, Ge J, Guo Y, Sun Y, Liu M, Xiao H (2014). MiR-203 is downregulated in laryngeal squamous cell carcinoma and can suppress proliferation and induce apoptosis of tumours. Tumour biology.

[23] Bian K, Fan J, Zhang X, Yang XW, Zhu HY, Wang L, Sun JY, Meng YL, Cui PC, Cheng SY, Zhang J, Zhao J, Yang AG, Zhang R (2012). MicroRNA-203 leads to G1 phase cell cycle arrest in laryngeal carcinoma cells by directly targeting survivin. FEBS letters.

[24] Hou B, Ishinaga H, Midorikawa K, Shah SA, Nakamura S, Hiraku Y, Oikawa S, Murata M, Takeuchi K (2015). Circulating microRNAs as novel prognosis biomarkers for head and neck squamous cell carcinoma. Cancer biology & therapy.

[25] Yan B, Fu Q, Lai L, Tao X, Fei Y, Shen J, Chen Z, Wang Q (2012). Downregulation of microRNA 99a in oral squamous cell carcinomas contributes to the growth and survival of oral cancer cells. Molecular Medicine Reports.

[26] Doghman M, El Wakil A, Cardinaud B, Thomas E, Wang JL, Zhao W, Peralta-Del Valle MHC, Figueiredo BC, Zambetti GP, Lalli E (2010). Regulation of Insulin-like Growth Factor-Mammalian Target of Rapamycin Signaling by MicroRNA in Childhood Adrenocortical Tumors. Cancer Res.

[27] Chen ZJ, Jin Y, Yu DS, Wang AX, Mahjabeen I, Wang C, Liu XQ, Zhou XF (2012). Down-regulation of the microRNA-99 family members in head and neck squamous cell carcinoma. Oral Oncol.

[28] Lara PC, Bordon E, Rey A, Moreno M, Lloret M, Henriquez-Hernandez LA (2011). IGF-1R expression predicts clinical outcome in patients with locally advanced oral squamous cell carcinoma. Oral Oncol.

[29] Mitra S, Mukherjee N, Das S, Das P, Panda CK, Chakrabarti J (2014). Anomalous altered expressions of downstream gene-targets in TP53-miRNA pathways in head and neck cancer. Scientific reports.

[30] Zhao B, Han H, Chen J, Zhang Z, Li S, Fang F, Zheng Q, Ma Y, Zhang J, Wu N, Yang Y (2014). MicroRNA let-7c inhibits migration and invasion of human non-small cell lung cancer by targeting ITGB3 and MAP4K3. Cancer letters.

[31] Kikkawa N, Hanazawa T, Fujimura L, Nohata N, Suzuki H, Chazono H, Sakurai D, Horiguchi S, Okamoto Y, Seki N (2010). miR-489 is a tumour-suppressive miRNA target PTPN11 in hypopharyngeal squamous cell carcinoma (HSCC). Br J Cancer.

[32] Hui AB, Lenarduzzi M, Krushel T, Waldron L, Pintilie M, Shi W, Perez-Ordonez B, Jurisica I, O'sullivan B, Waldron J, Gullane P, Cummings B, Liu FF (2010). Comprehensive MicroRNA profiling for head and neck squamous cell carcinomas. Clinical cancer research.

[33] Zhi X, Lamperska K, Golusinski P, Schork NJ, Luczewski L, Kolenda T, Golusinski W, Masternak MM (2015). Gene expression analysis of head and neck squamous cell carcinoma survival and recurrence. Oncotarget.

[34] Leelahavanichkul K, Amornphimoltham P, Molinolo AA, Basile JR, Koontongkaew S, Gutkind JS (2014). A role for p38 MAPK in head and neck cancer cell growth and tumor-induced angiogenesis and lymphangiogenesis. Mol Oncol.

[35] Shi Y, Massague J (2003). Mechanisms of TGF-beta signaling from cell membrane to the nucleus. Cell.

[36] Martin D, Abba MC, Molinolo AA, Vitale-Cross L, Wang ZY, Zaida M, Delic NC, Samuels Y, Lyons JG, Gutkind JS (2014). The head and neck cancer cell oncogenome: a platform for the development of precision molecular therapies. Oncotarget.

[37] Yoshino H, Chiyomaru T, Enokida H, Kawakami K, Tatarano S, Nishiyama K, Nohata N, Seki N, Nakagawa M (2011). The tumour-suppressive function of miR-1 and miR-133a targeting TAGLN2 in bladder cancer. British Journal of Cancer.

[38] Robinson MD, Oshlack A (2010). A scaling normalization method for differential expression analysis of RNA-seq data. Genome biology.

[39] McCarthy DJ, Chen Y, Smyth GK (2012). Differential expression analysis of multifactor RNA-Seq experiments with respect to biological variation. Nucleic acids research.

[40] Robinson MD, McCarthy DJ, Smyth GK (2010). edgeR: a Bioconductor package for differential expression analysis of digital gene expression data. Bioinformatics.

[41] Bair E, Tibshirani R (2004). Semi-supervised methods to predict patient survival from gene expression data. PLoS Biol.

[42] Huang da W, Sherman BT, Lempicki RA (2009). Systematic and integrative analysis of large gene lists using DAVID bioinformatics resources. Nature protocols.

