# Supplementary material for: Identification of a six microRNA signature as a novel potential prognostic biomarker in patients with head and neck squamous cell carcinoma
Source: Oncotarget. 2016 Feb 27;7(16):21579–90. doi: 10.18632/oncotarget.7781 (PMC5008307; doi:10.18632/oncotarget.7781)
Supplement: Supplementary file 1 [file oncotarget-07-21579-s001.pdf]

## SUPPLEMENTARY FIGURES AND TABLES

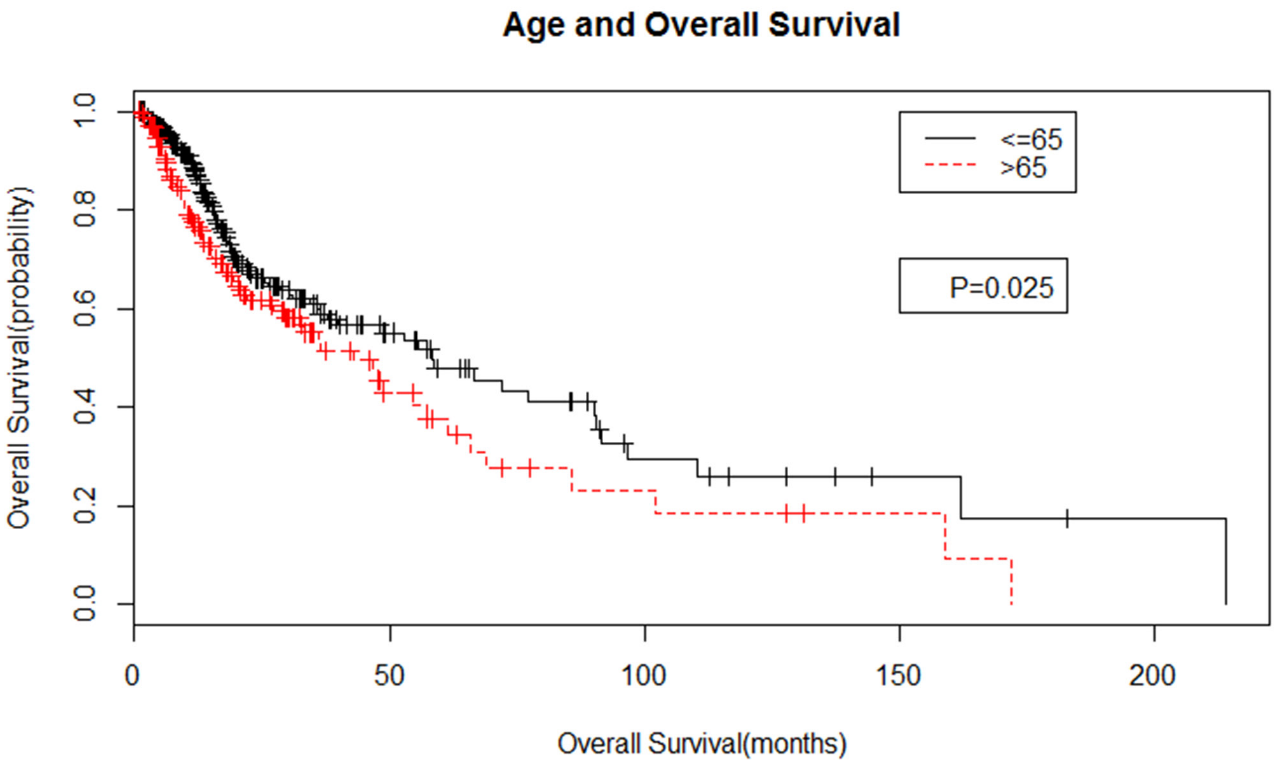


**Supplementary Figure S1: Kaplan-Meier survival estimates by age in HNSCC.** The P-value of overall log-rank test was 0.025.

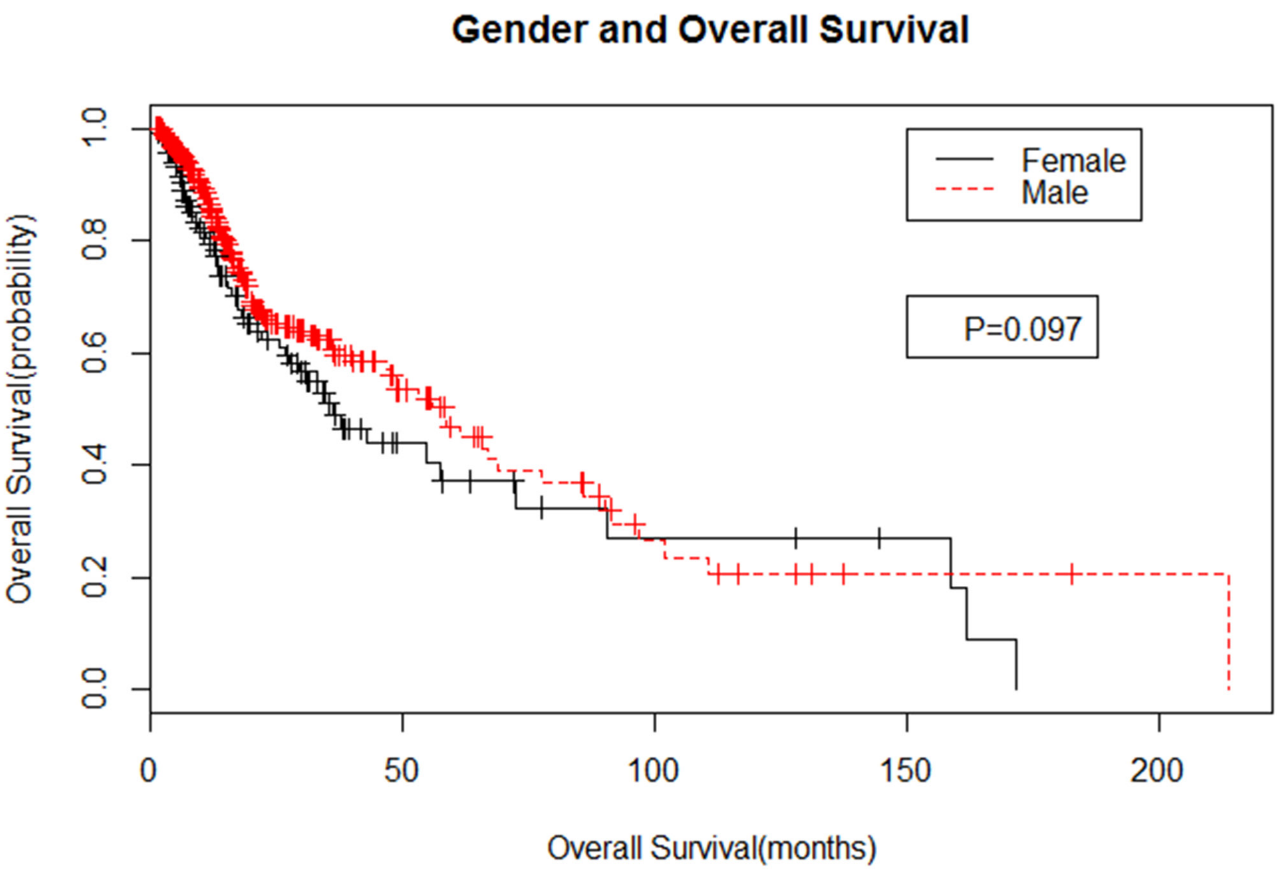


**Supplementary Figure S2: Kaplan-Meier survival estimates by gender in HNSCC.** The P-value of overall log-rank test was 0.10.

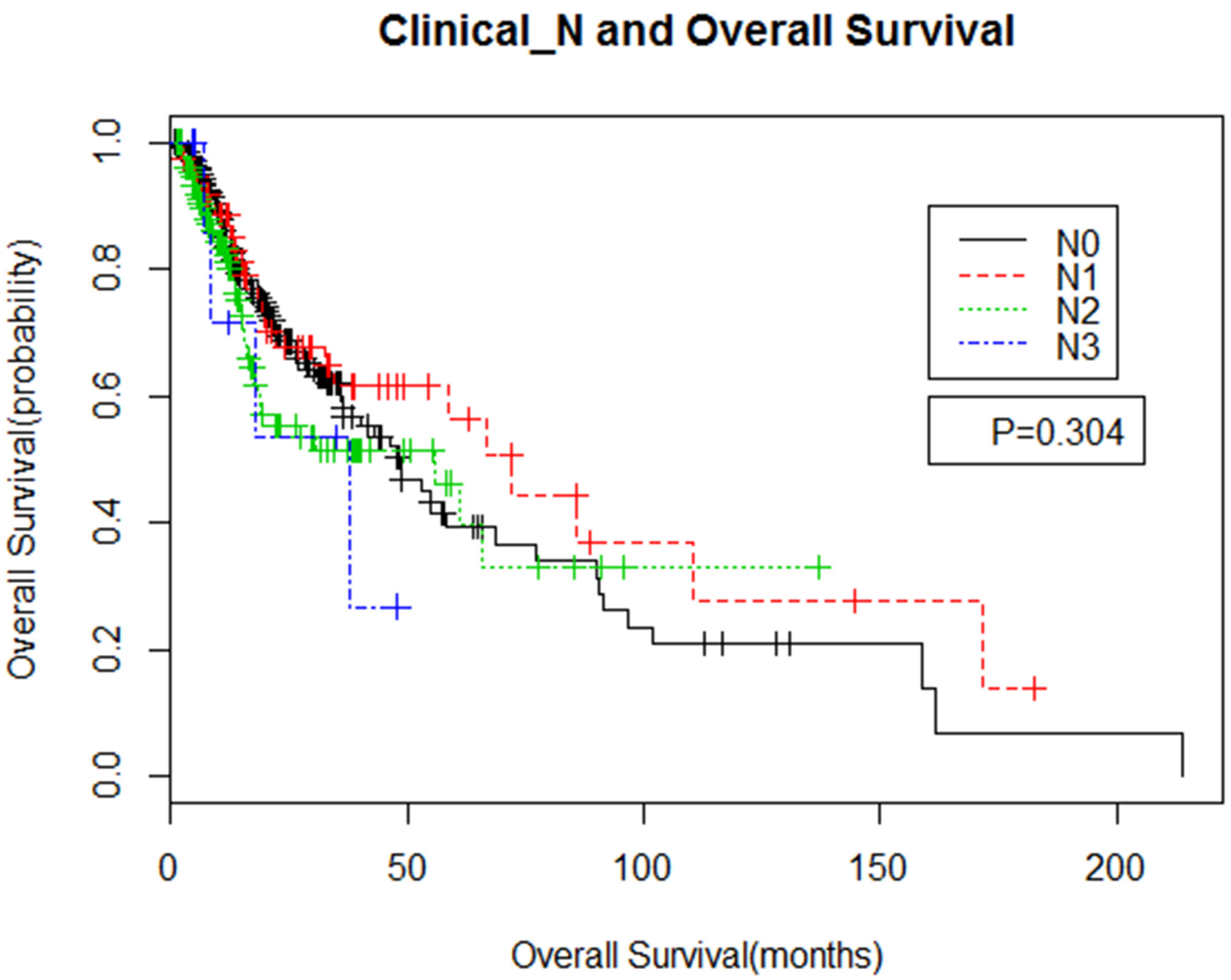


**Supplementary Figure S3: Kaplan-Meier survival estimates by lymph node involvement (clinical_N) in HNSCC.** The P-value of overall log-rank test was 0.30.

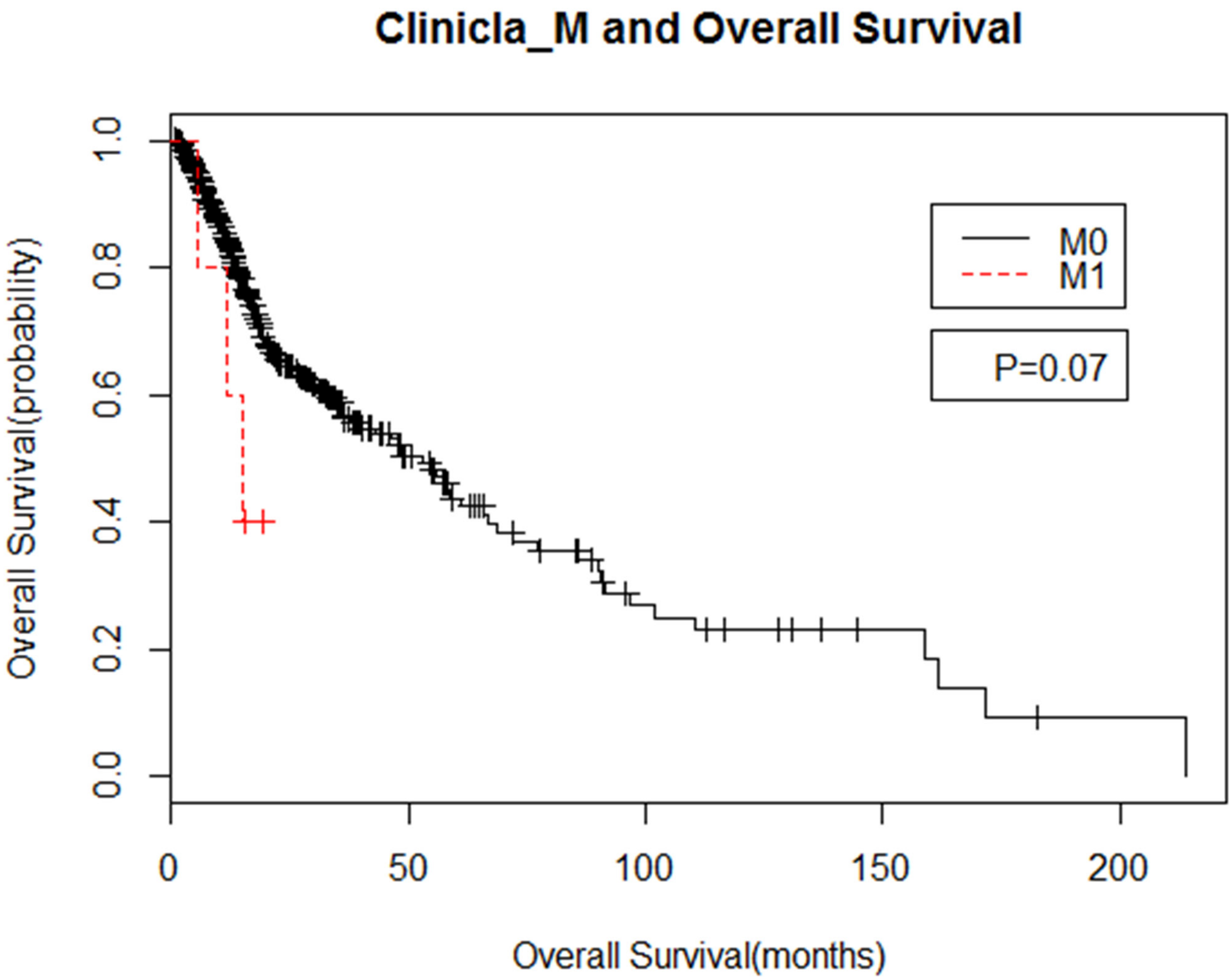


**Supplementary Figure S4: Kaplan-Meier survival estimates by distant metastases (clinical_M) in HNSCC.** The P-value of overall log-rank test was 0.07.

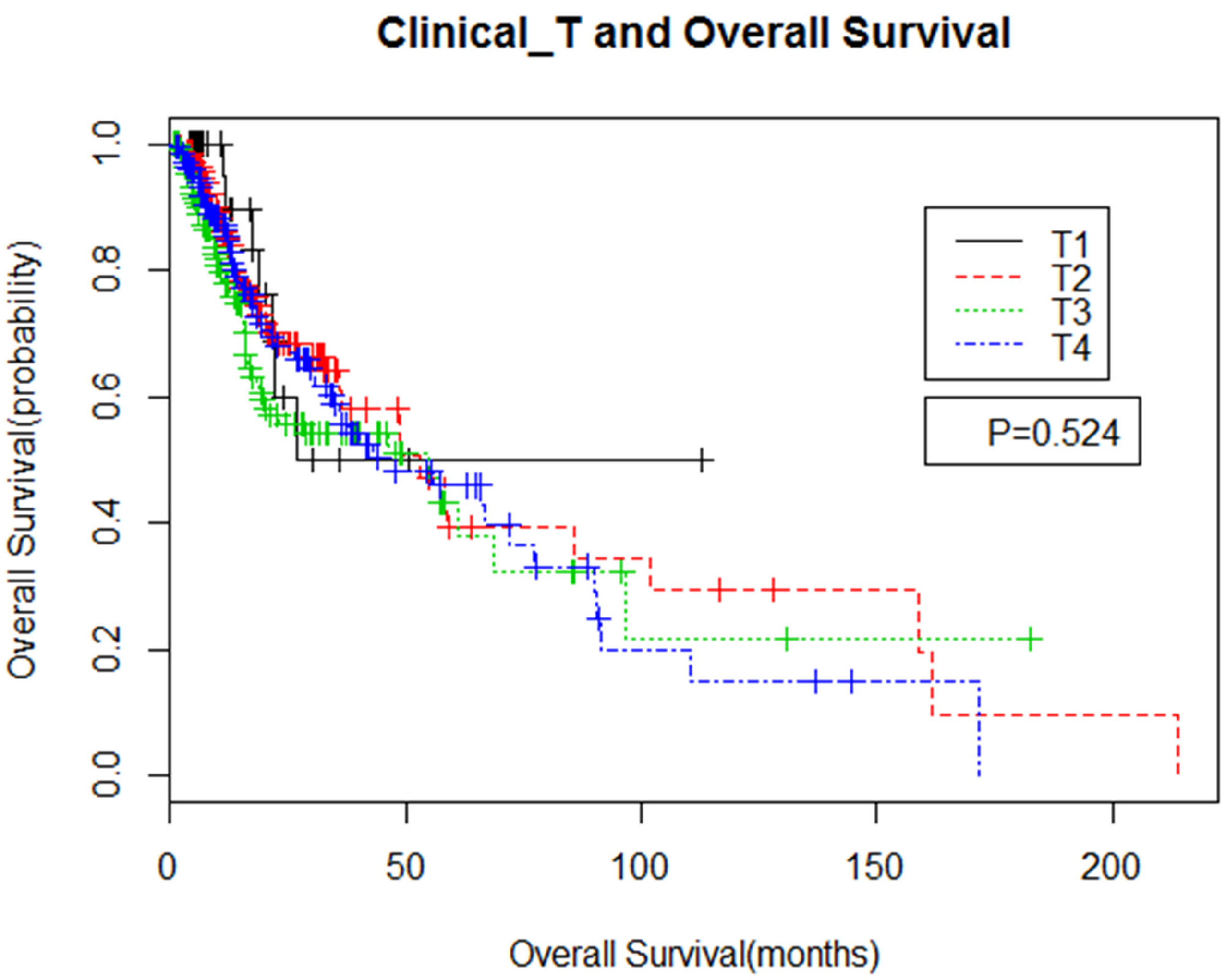


**Supplementary Figure S5: Kaplan-Meier survival estimates by primary tumor (clinical_T) in HNSCC.** The P-value of overall log-rank test was 0.52.

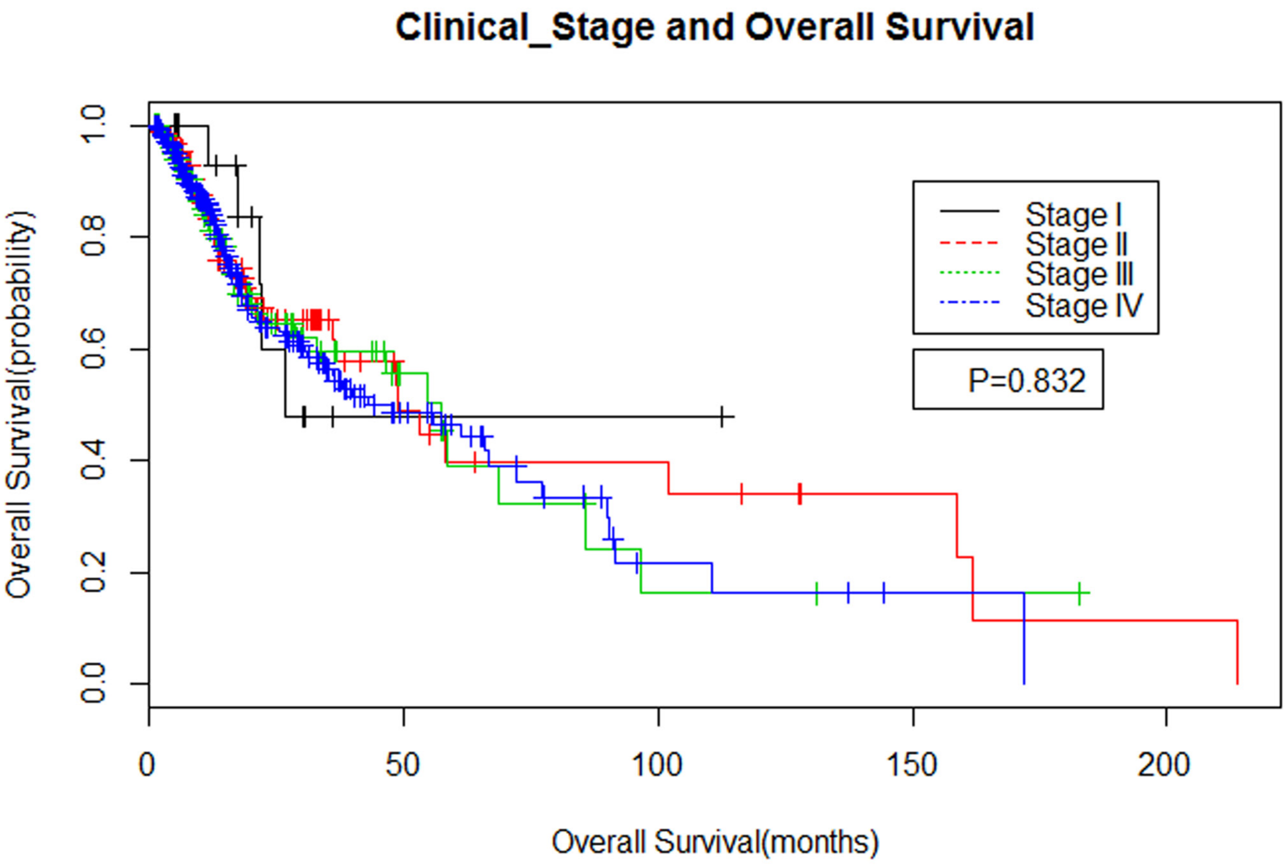


**Supplementary Figure S6: Kaplan-Meier survival estimates by disease stage (clinical_stage) in HNSCC.** The P-value of overall log-rank test was 0.83.

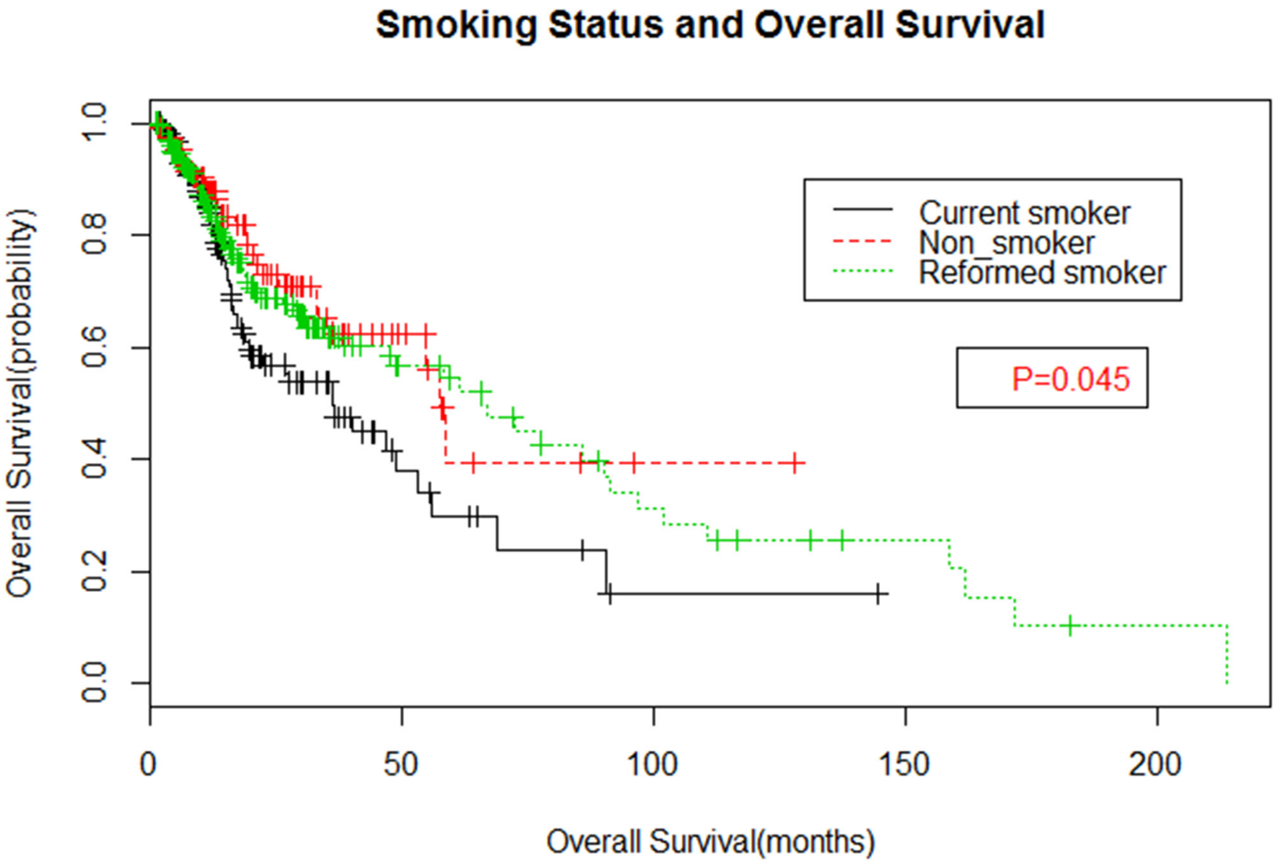


**Supplementary Figure S7: Kaplan-Meier survival estimates by smoking status in HNSCC.** The P-value of overall log-rank test was 0.045.

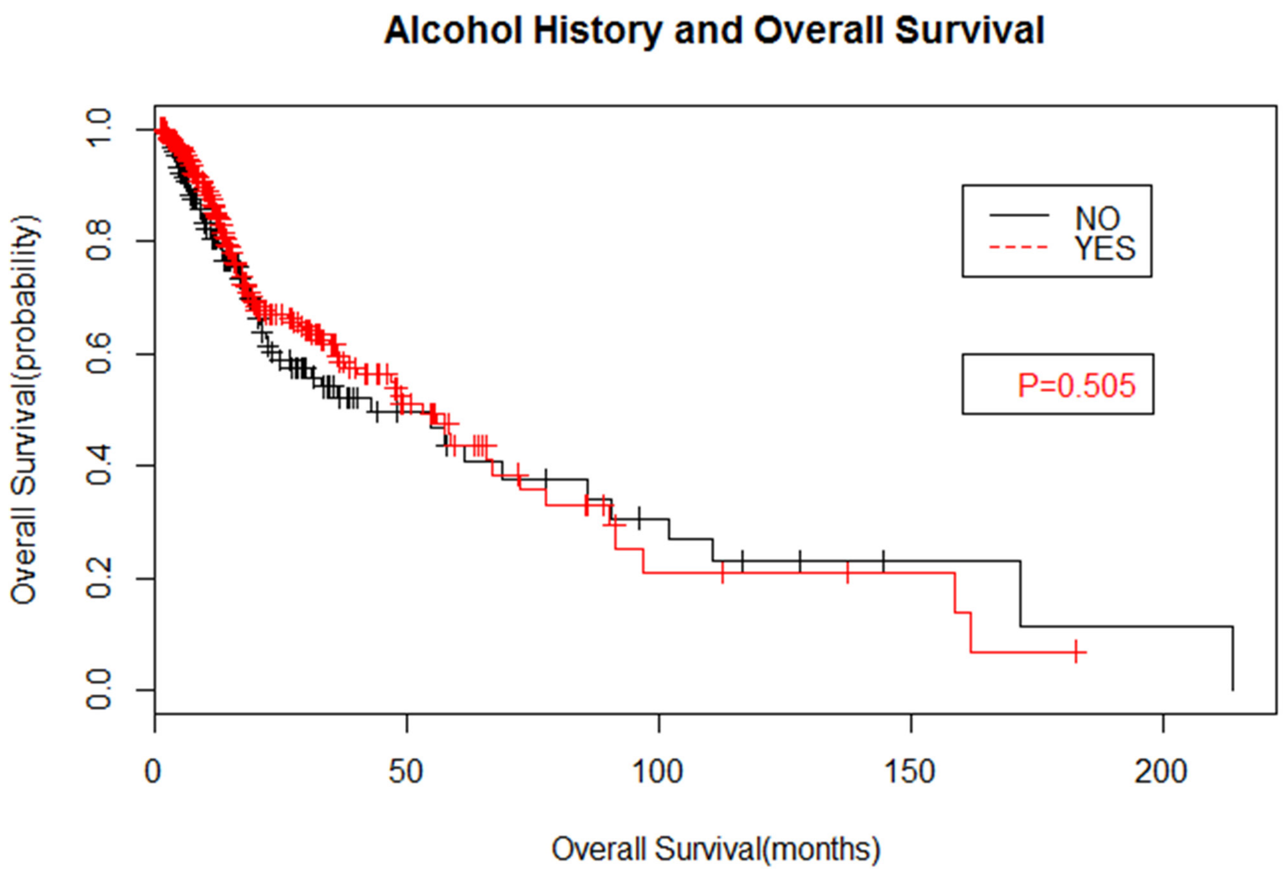


**Supplementary Figure S8: Kaplan-Meier survival estimates by alcohol history in HNSCC.** The P-value of overall log-rank test was 0.505.

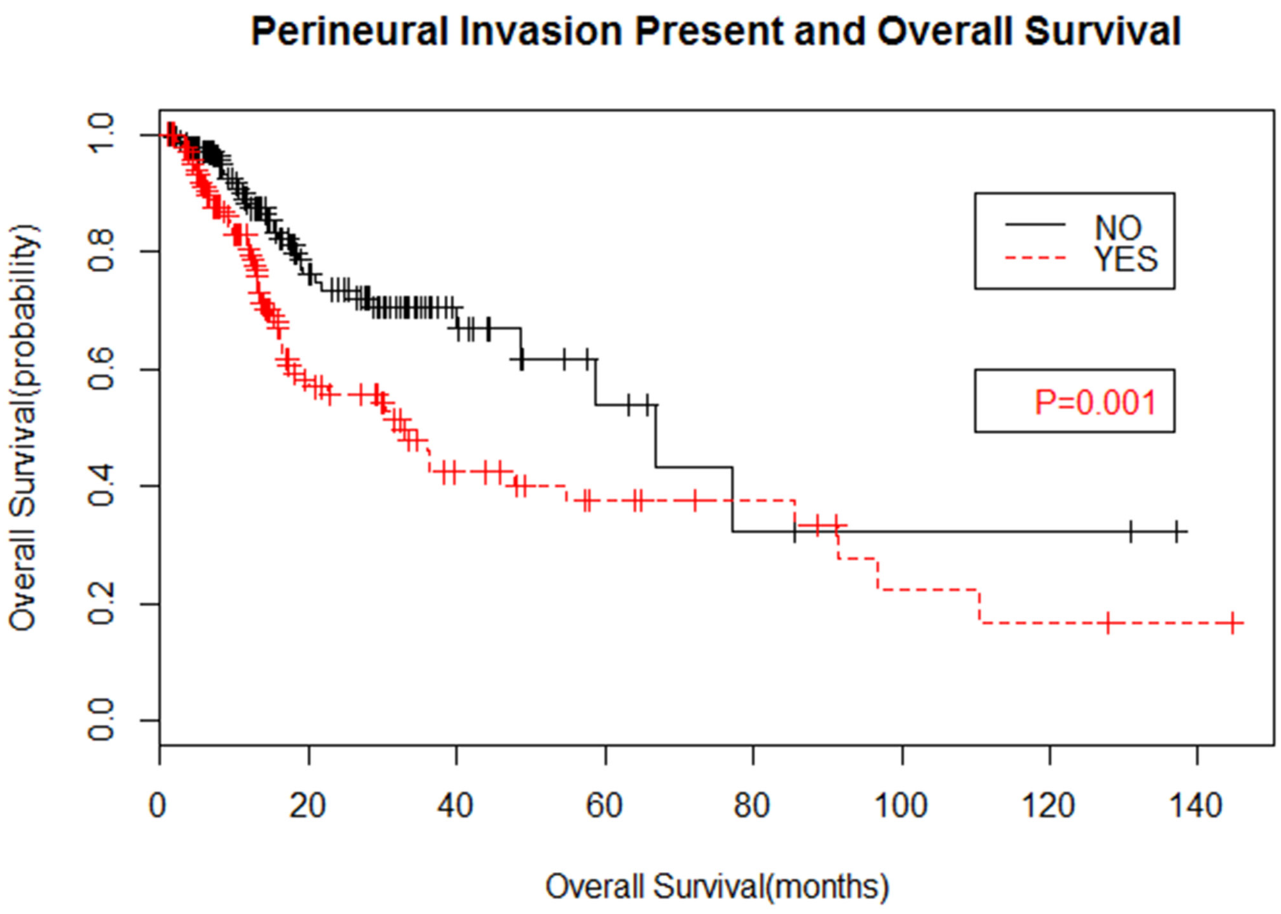


**Supplementary Figure S9: Kaplan-Meier survival estimates by perineural invasion present in HNSCC.** The P-value of overall log-rank test was 0.001.

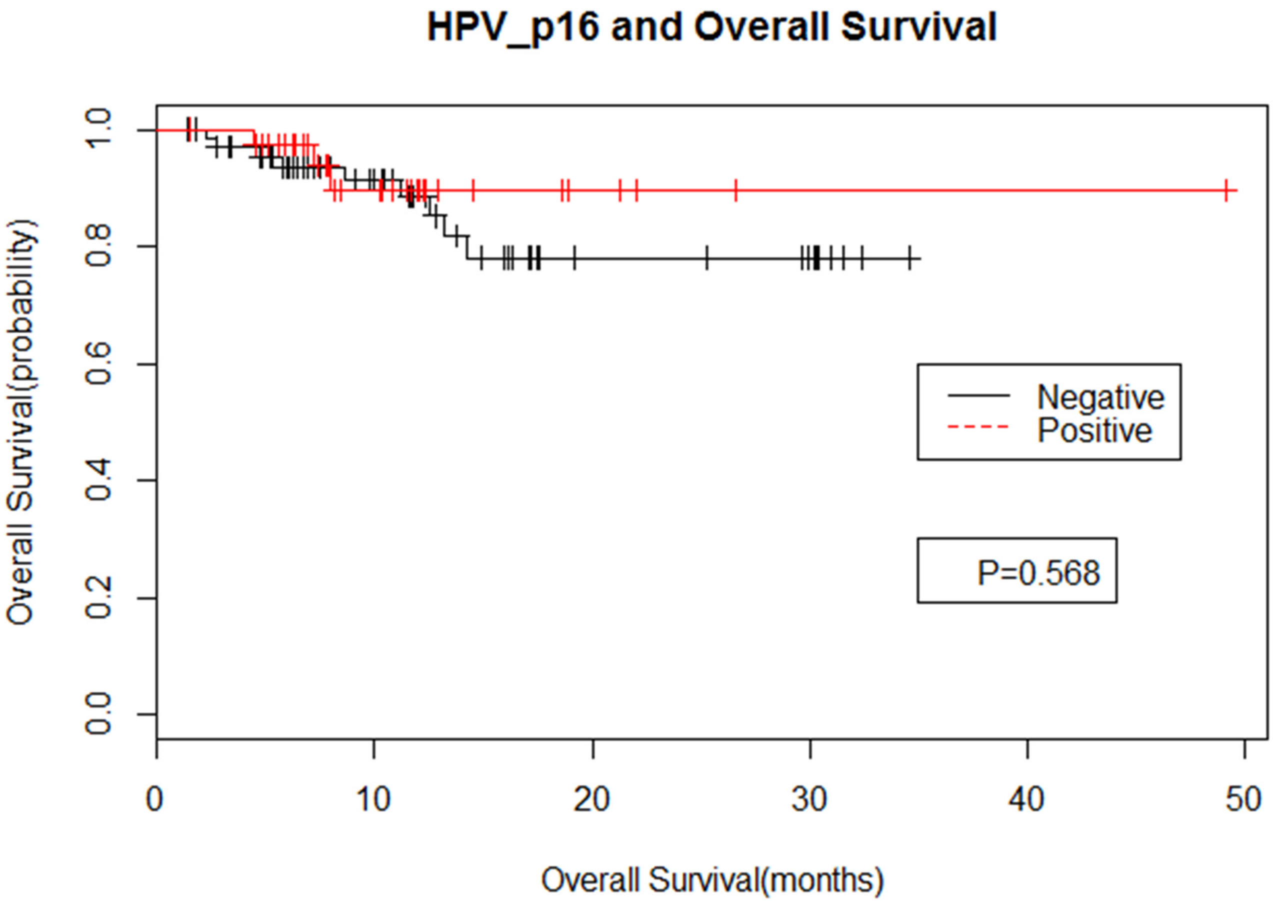


**Supplementary Figure S10: Kaplan-Meier survival estimates by HPV_p16 status in HNSCC.** The P-value of overall log-rank test was 0.57.

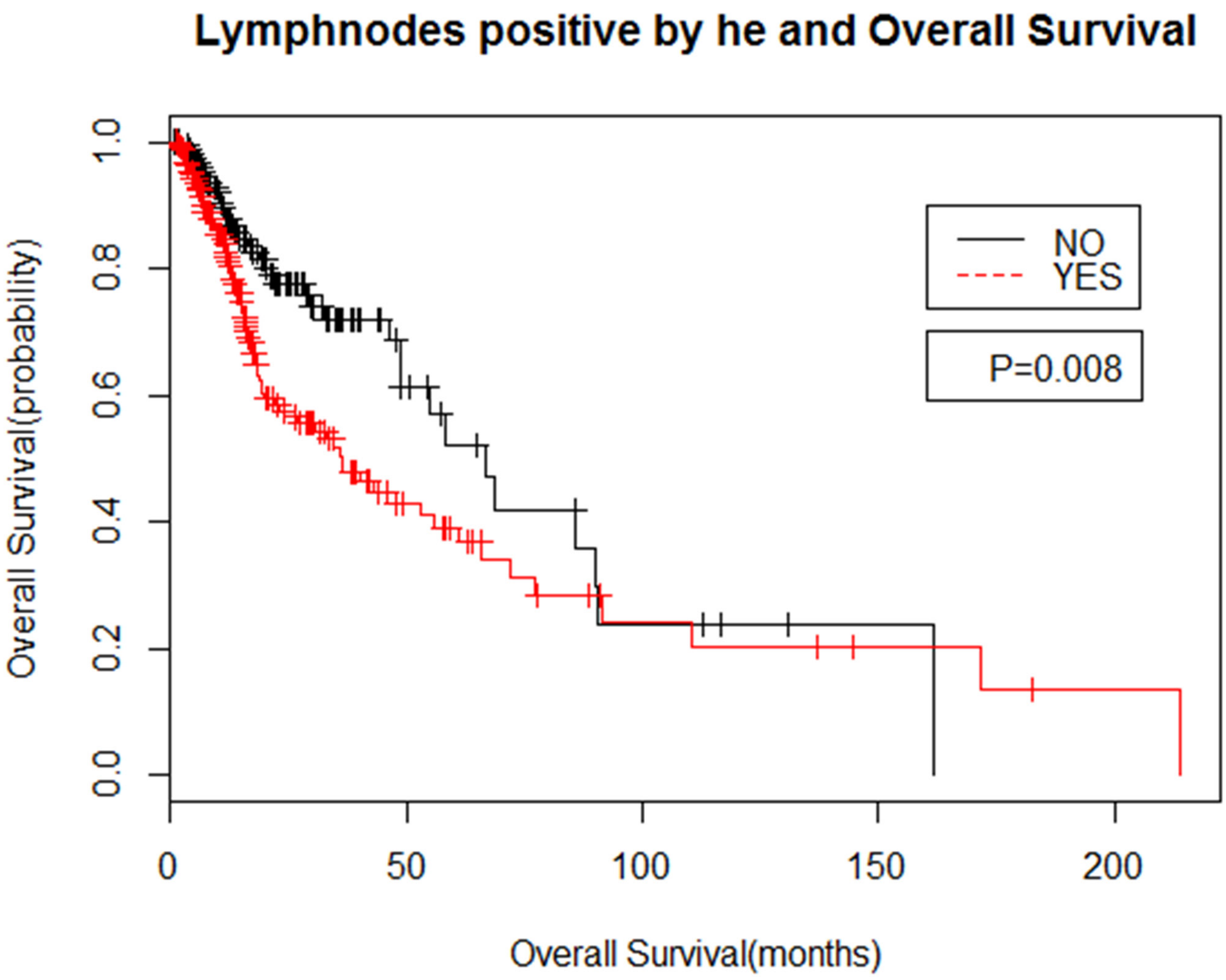


**Supplementary Figure S11: Kaplan-Meier survival estimates by lympnodes positive by he in HNSCC.** The P-value of overall log-rank test was 0.008.

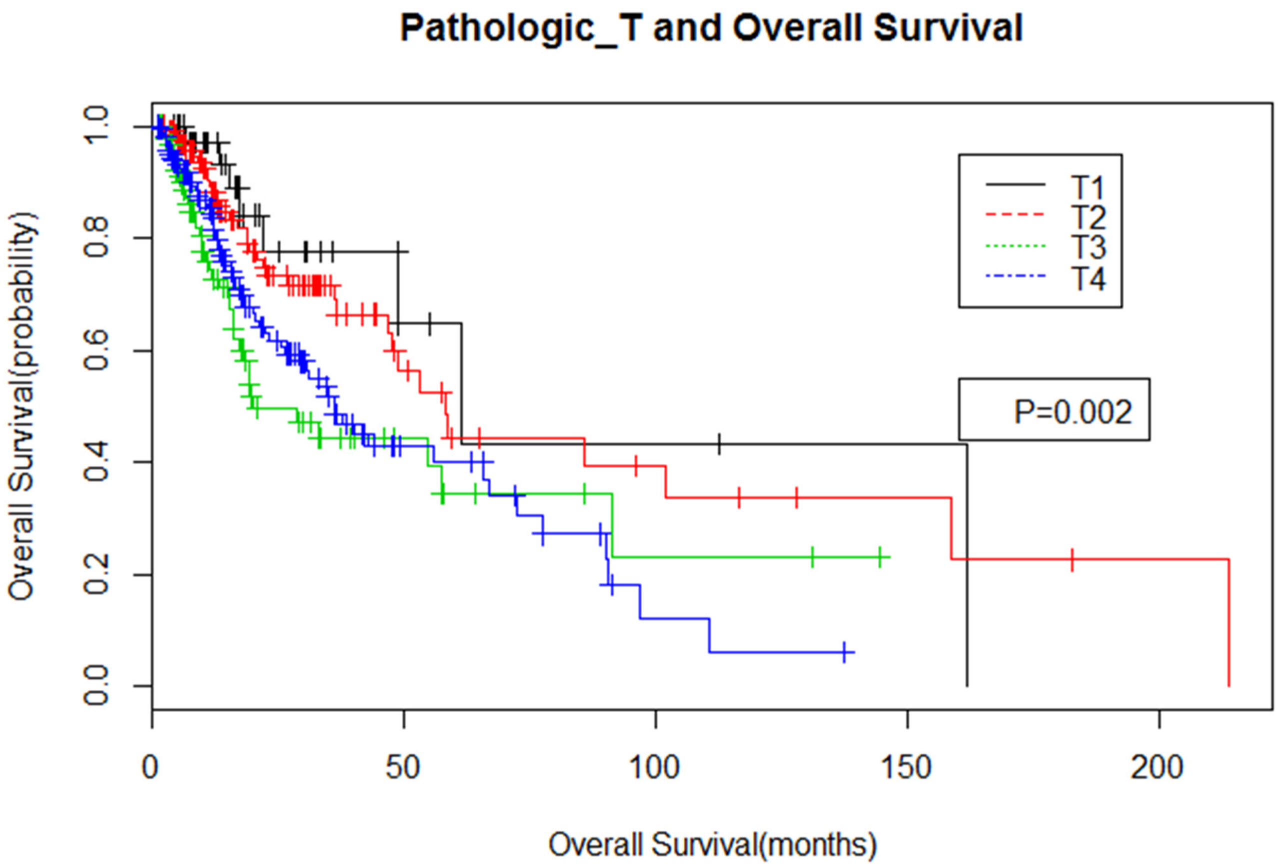


**Supplementary Figure S12: Kaplan-Meier survival estimates by pathologic T in HNSCC.** The P-value of overall log-rank test was 0.002.

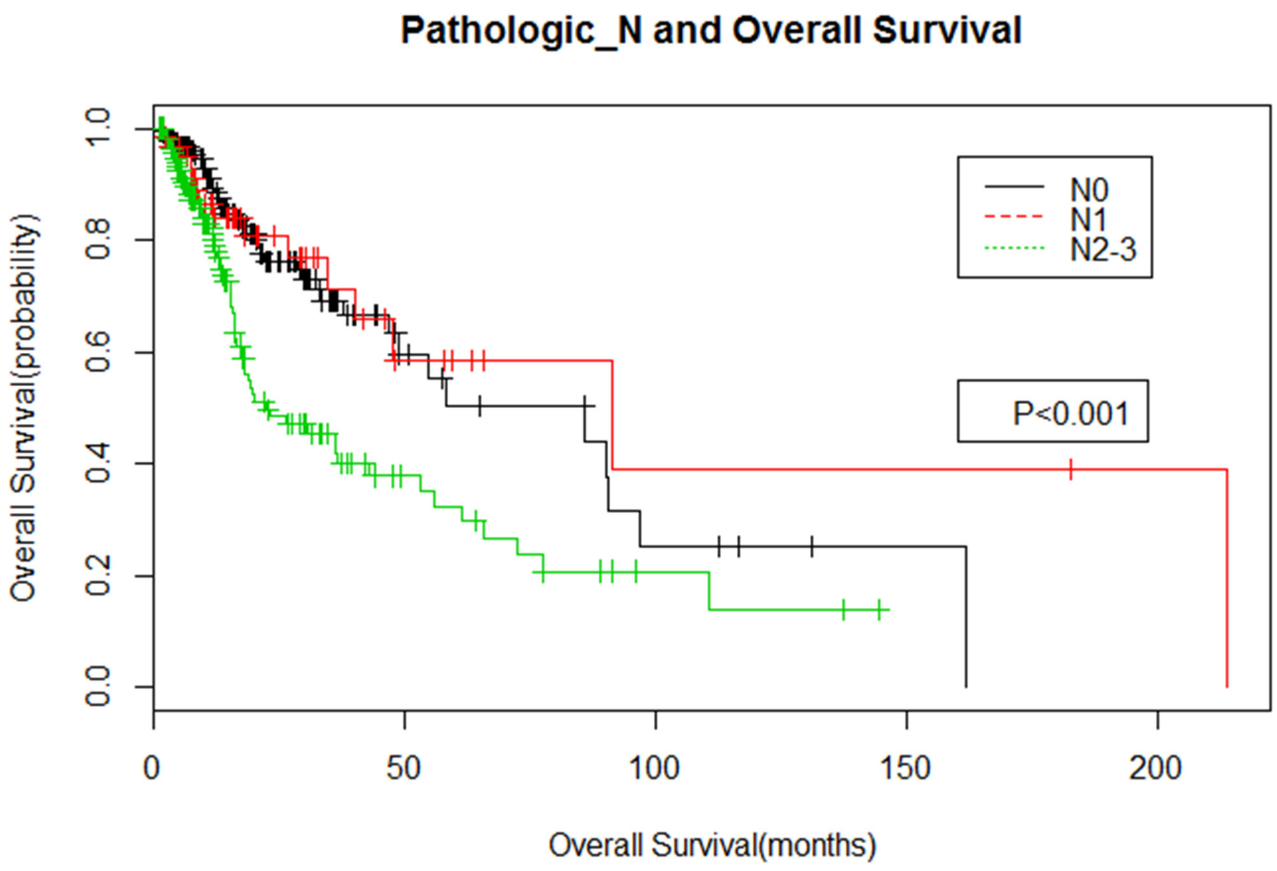


**Supplementary Figure S13: Kaplan-Meier survival estimates by pathologic N in HNSCC.** The P-value of overall log-rank test was <0.001.

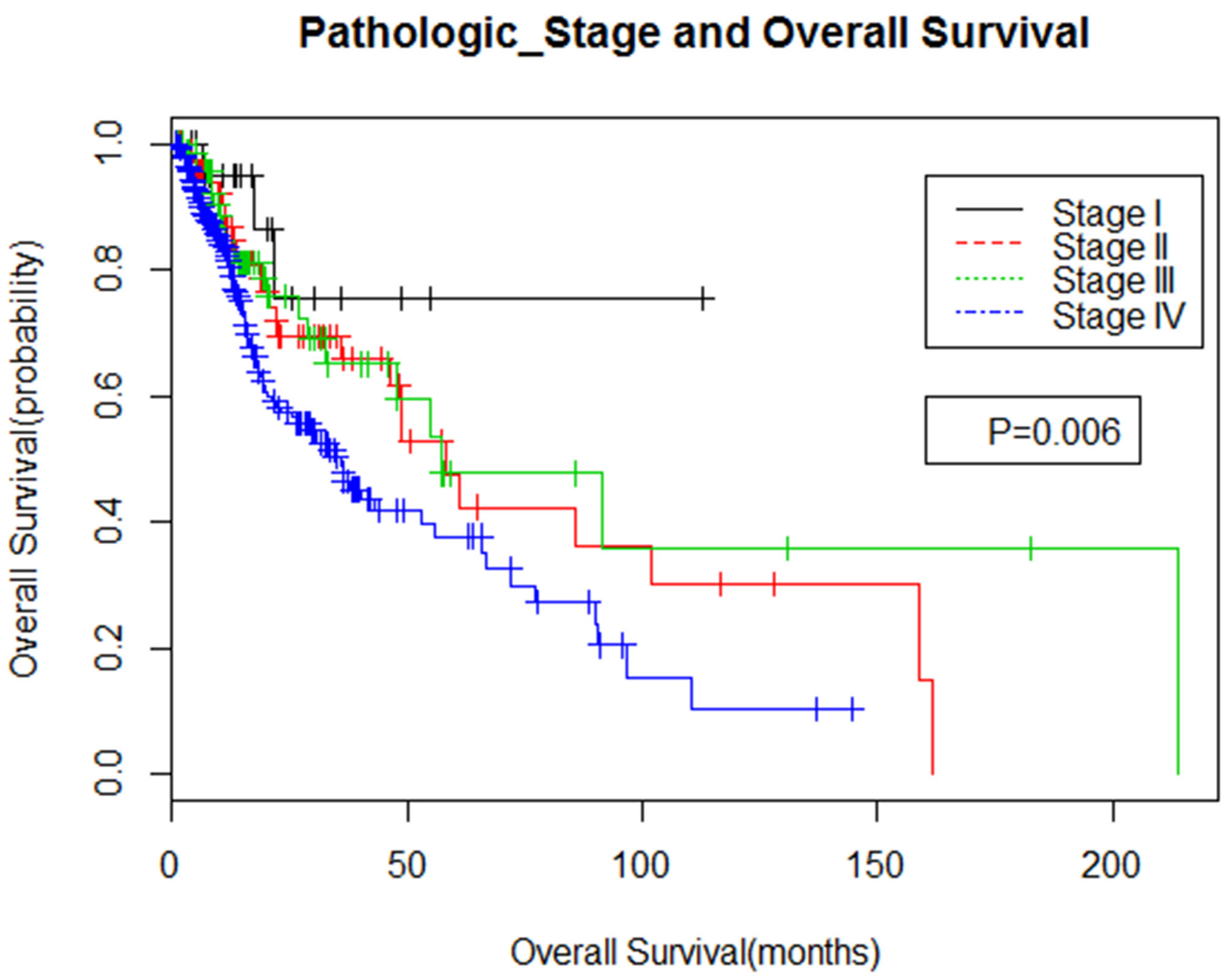


**Supplementary Figure S14: Kaplan-Meier survival estimates by pathologic stage in HNSCC.** The P-value of overall log-rank test was 0.006.

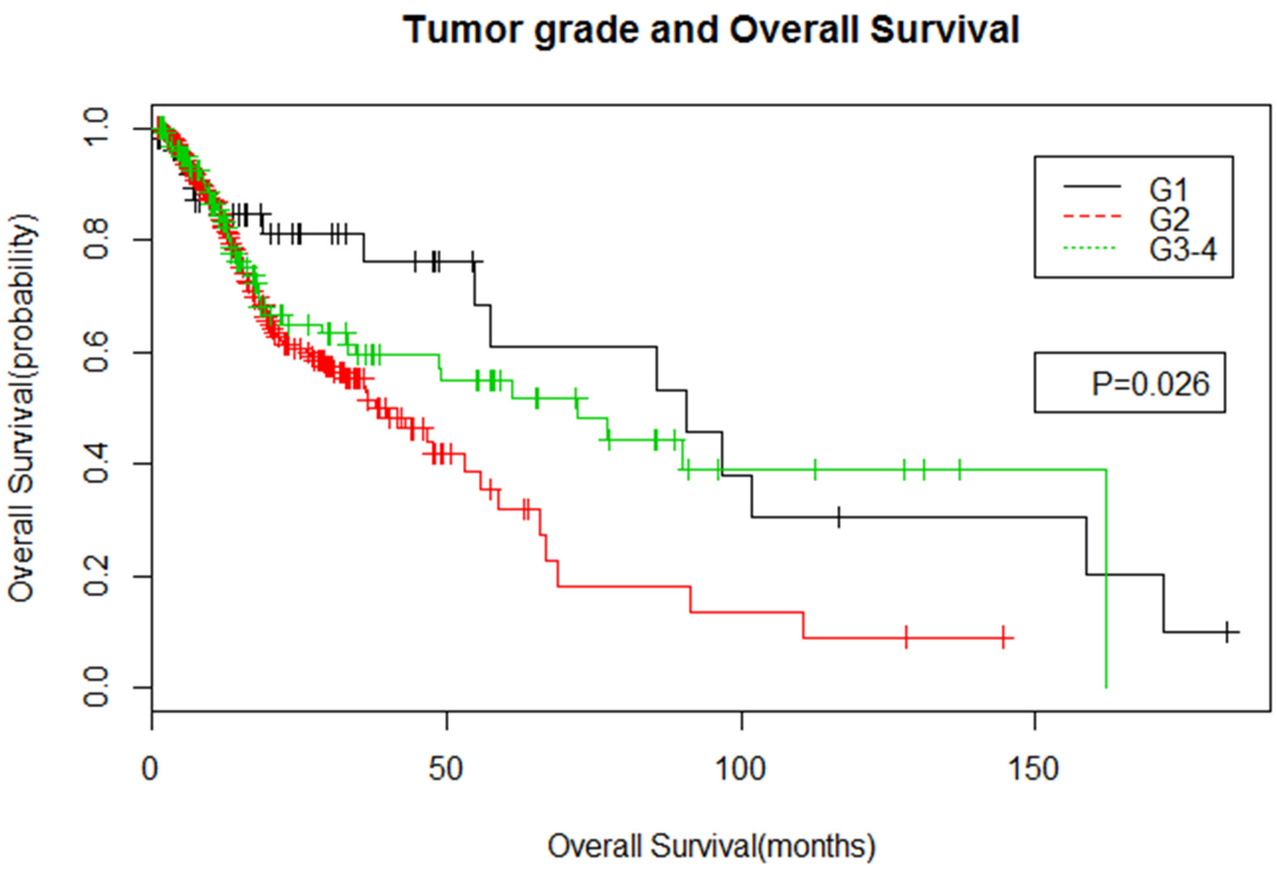


**Supplementary Figure S15: Kaplan-Meier survival estimates by tumor grade in HNSCC.** The P-value of overall log-rank test was 0.026.

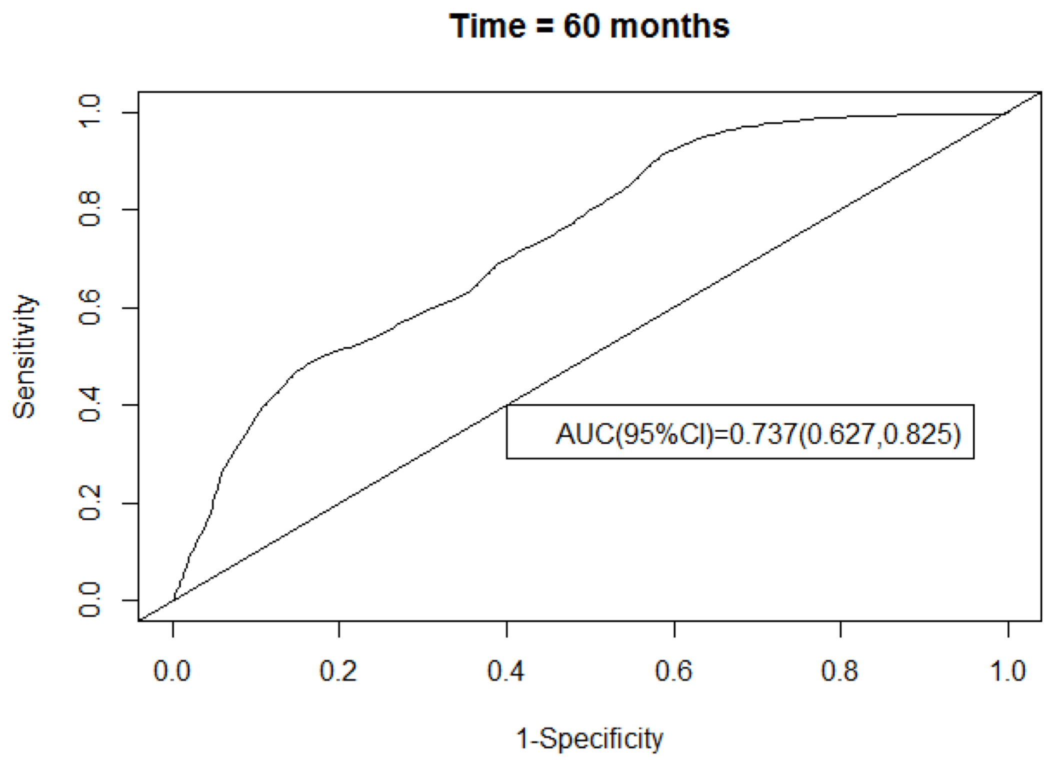


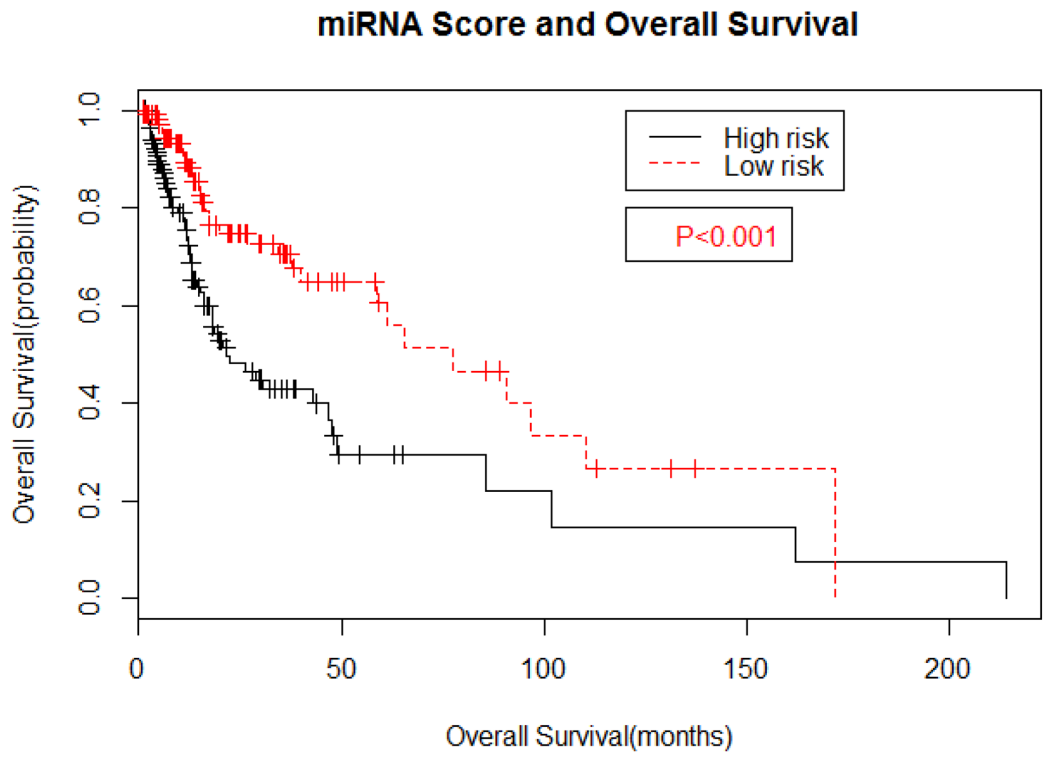


**Supplementary Figure S16: Kaplan–Meier and ROC curves for the six microRNA signature in TCGA HNSCC testing set. A.** The ROC curve for predicting 5-year survival had an AUC of 0.737 (95%CI, 0.627-0.825). **B.** The Kaplan–Meier curves for HNSCC risk groups obtained from the TCGA cohort testing set (n = 246) divided by the cutoff point. The P value of the log-rank test was <0.01.

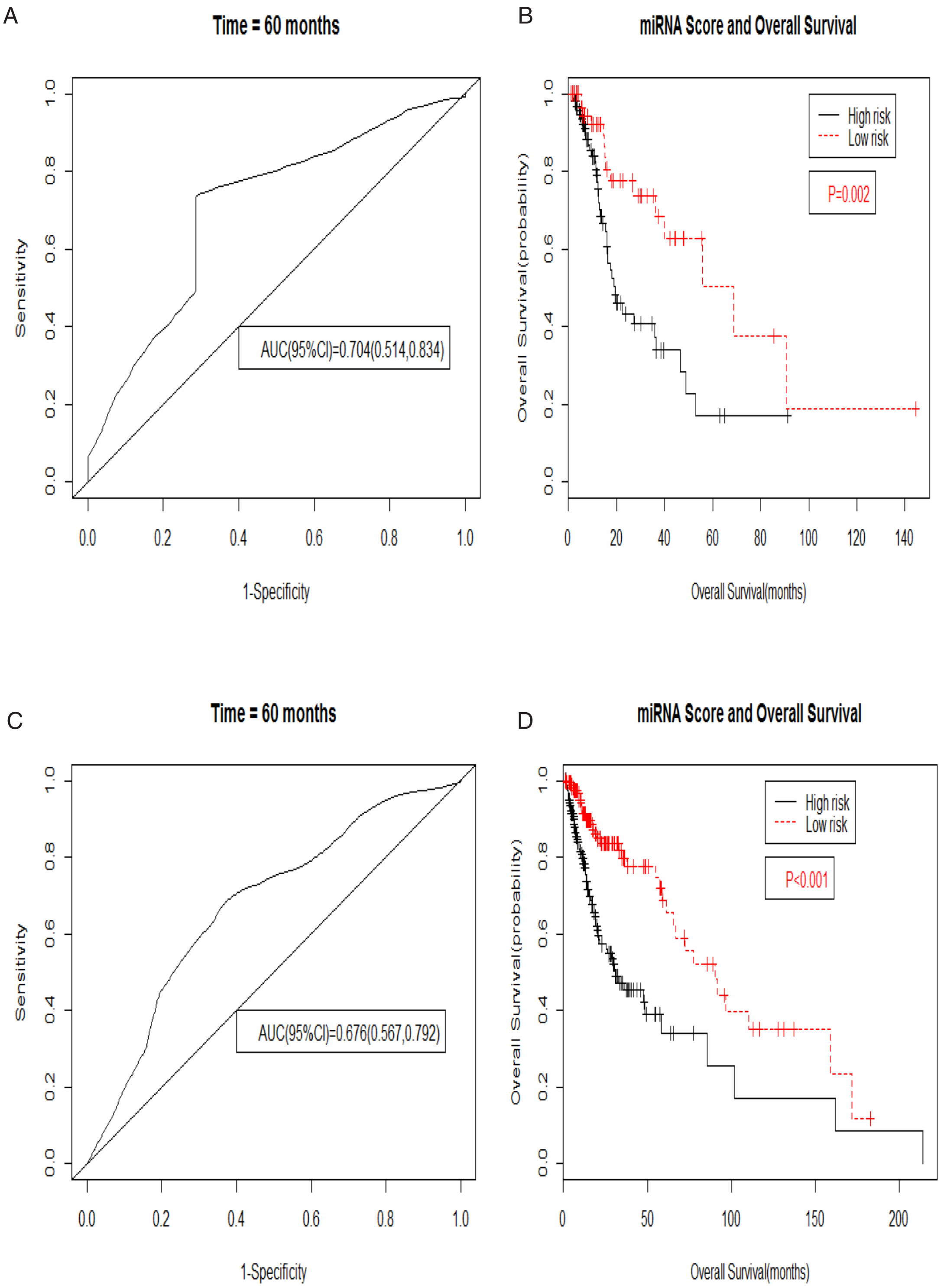


**Supplementary Figure S17: Kaplan–Meier and ROC curves for the six microRNA signature in TCGA HNSCC smoking group. A.** The ROC curve for predicting 5-year survival had an AUC of 0.704 (95%CI, 0.514-0.834) in the current smoking group. **B.** The Kaplan–Meier curves for HNSCC risk groups obtained from the TCGA cohort current smoking group divided by the cutoff point. The P value of the log-rank test was <0.01. **C.** The ROC curve for predicting 5-year survival had an AUC of 0.676 (95%CI, 0.567-0.792) in the current non_smoking group. **D.** The Kaplan–Meier curves for HNSCC risk groups obtained from the TCGA cohort current non_smoking group divided by the cutoff point. The P value of the log-rank test was <0.01.

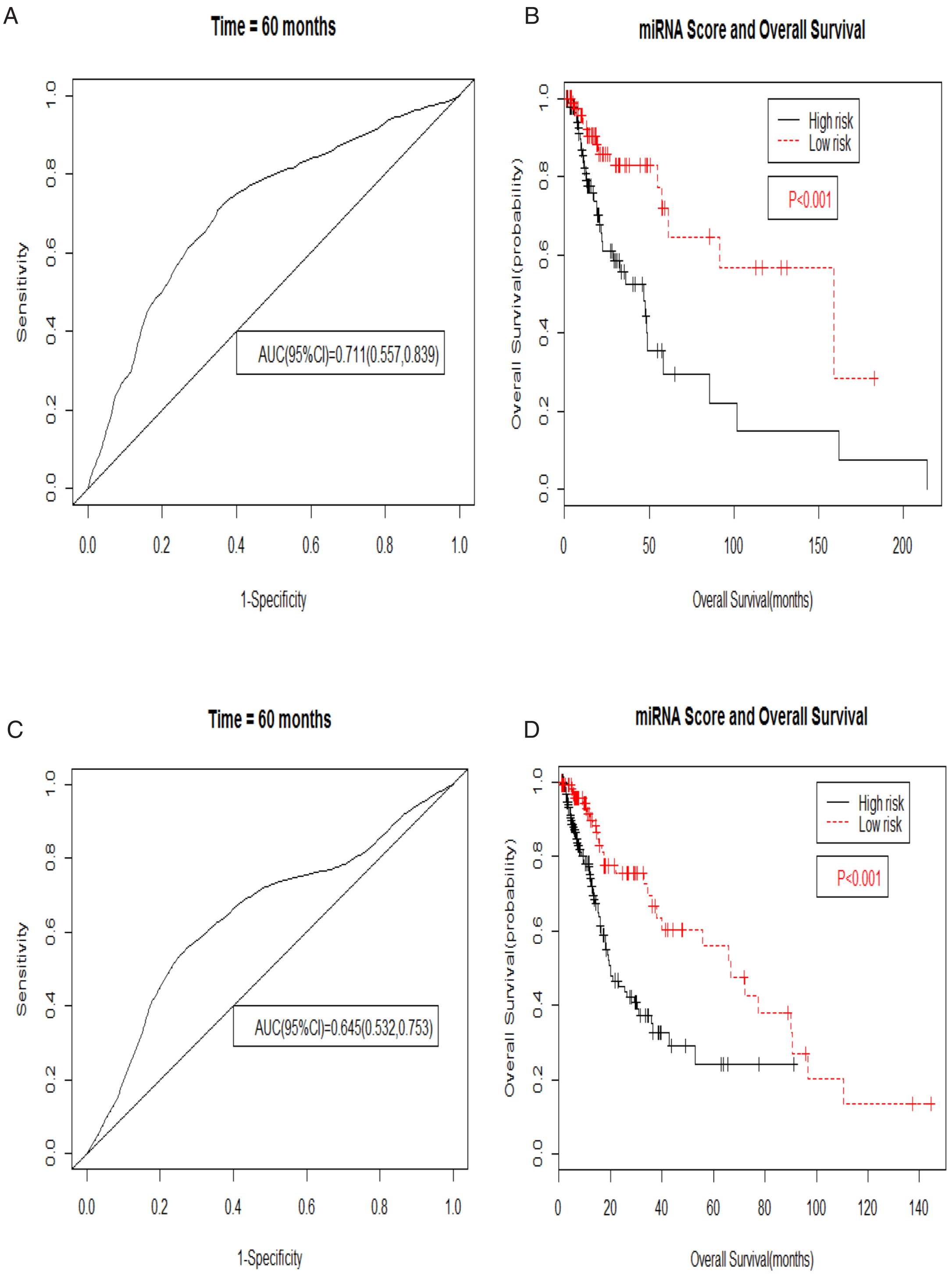


**Supplementary Figure S18: Kaplan–Meier and ROC curves for the six microRNA signature in TCGA HNSCC pathologic stage group. A.** The ROC curve for predicting 5-year survival had an AUC of 0.711 (95%CI, 0.557-0.839) in the pathologic stage I-III group. **B.** The Kaplan–Meier curves for HNSCC risk groups obtained from the TCGA cohort pathologic stage I-III group divided by the cutoff point. The P value of the log-rank test was <0.01. **C.** The ROC curve for predicting 5-year survival had an AUC of 0.654 (95%CI, 0.532-0.753) in the pathologic stage IV group. **D.** The Kaplan–Meier curves for HNSCC risk groups obtained from the TCGA cohort pathologic stage IV group divided by the cutoff point. The P value of the log-rank test was <0.01.

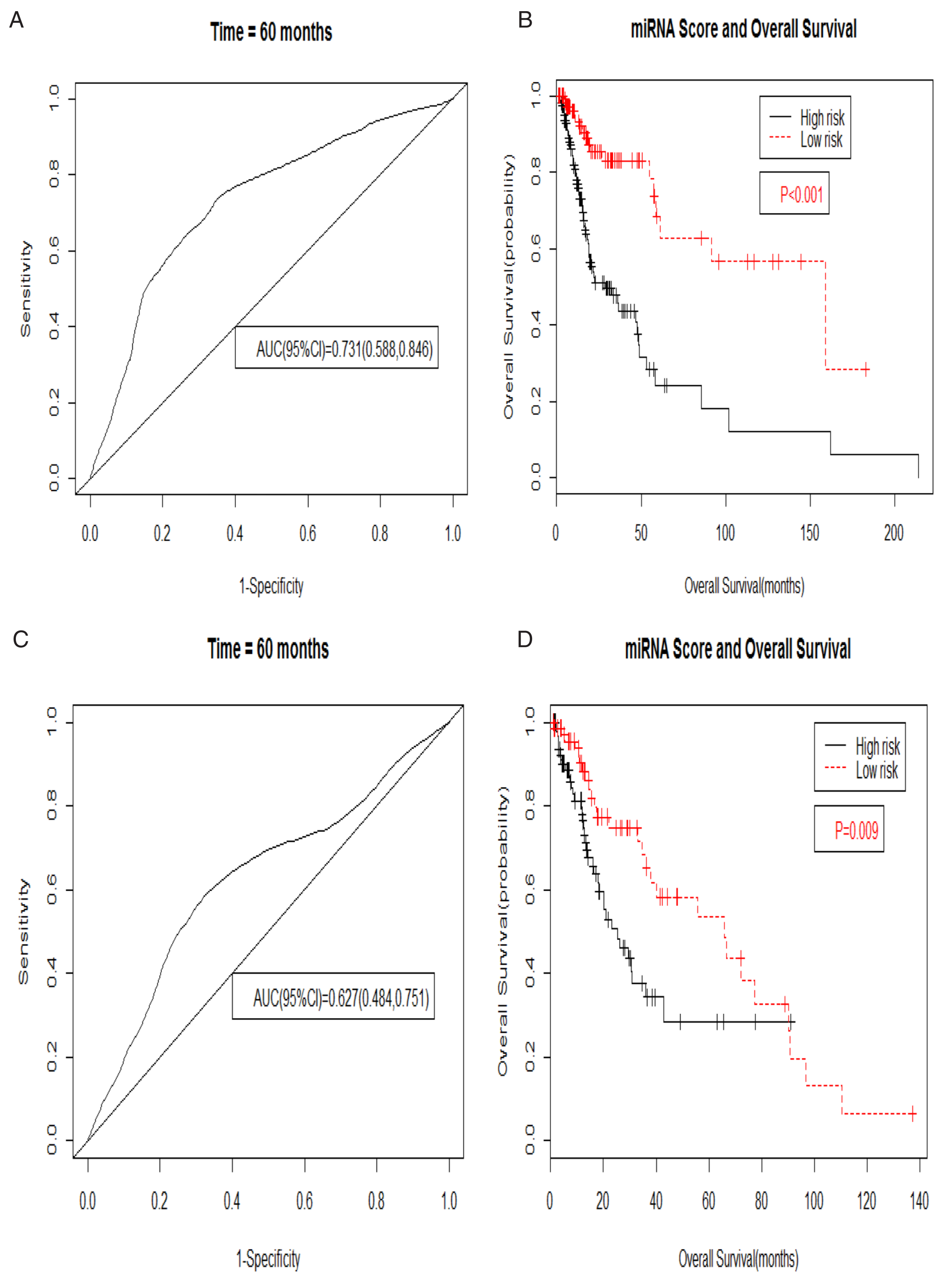


**Supplementary Figure S19: Kaplan–Meier and ROC curves for the six microRNA signature in TCGA HNSCC pathologic T group. A.** The ROC curve for predicting 5-year survival had an AUC of 0.731 (95%CI, 0.588-0.846) in the pathologic T1-3 group. **B.** The Kaplan–Meier curves for HNSCC risk groups obtained from the TCGA cohort pathologic T1-3 group divided by the cutoff point. The P value of the log-rank test was <0.01. **C.** The ROC curve for predicting 5-year survival had an AUC of 0.627 (95%CI, 0.484-0.751) in the pathologic T4 group. **D.** The Kaplan–Meier curves for HNSCC risk groups obtained from the TCGA cohort pathologic T4 group divided by the cutoff point. The P value of the log-rank test was <0.01.

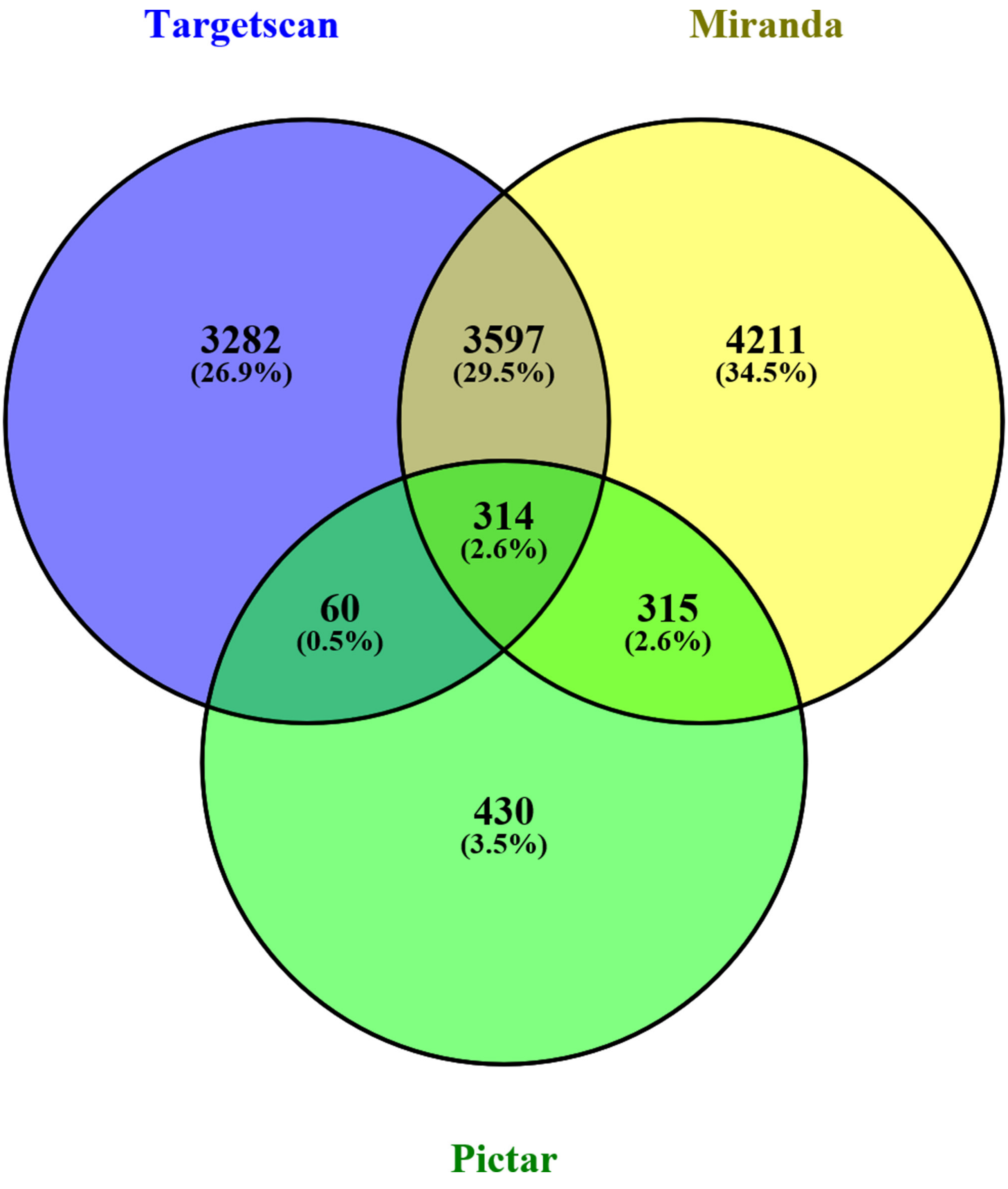


**Supplementary Figure S20: The Venny plot for selecting the target genes which it was in all of the three datasets.**

**Supplementary Table S1: The differentially expressed miRNAs in HNSCC and normal tissue**

**See Supplementary File 1**

**Supplementary Table S2: The enrich analysis for target genes of six microRNA signature**

**See Supplementary File 2**
